# Current State of Knowledge Regarding WHO High Priority Pathogens—Resistance Mechanisms and Proposed Solutions through Candidates Such as Essential Oils: A Systematic Review

**DOI:** 10.3390/ijms24119727

**Published:** 2023-06-04

**Authors:** Mirabela Romanescu, Camelia Oprean, Adelina Lombrea, Bianca Badescu, Ana Teodor, George D. Constantin, Minodora Andor, Roxana Folescu, Delia Muntean, Corina Danciu, Olivia Dalleur, Stefan Laurentiu Batrina, Octavian Cretu, Valentina Oana Buda

**Affiliations:** 1 Doctoral School, “Victor Babeş” University of Medicine and Pharmacy, 2 Eftimie Murgu Street, 300041 Timisoara, Romania; mirabela.romanescu@umft.ro (M.R.); adelina.lombrea@umft.ro (A.L.); bianca.badescu@medicis.ro (B.B.); ana.teodor@umft.ro (A.T.); george.constantin@umft.ro (G.D.C.); 2 Faculty of Medicine, “Victor Babeş” University of Medicine and Pharmacy, 2 Eftimie Murgu Street, 300041 Timisoara, Romania; andor.minodora@umft.ro (M.A.); folescu.roxana@umft.ro (R.F.); muntean.delia@umft.ro (D.M.);; 3 Faculty of Pharmacy, “Victor Babeş” University of Medicine and Pharmacy, 2 Eftimie Murgu Street, 300041 Timisoara, Romania; 4 OncoGen Centre, County Hospital ‘Pius Branzeu’, Blvd. Liviu Rebreanu 156, 300723 Timisoara, Romania; 5 Multidisciplinary Research Center on Antimicrobial Resistance, “Victor Babes” University of Medicine and Pharmacy, 2 Eftimie Murgu Square, 300041 Timisoara, Romania; 6 Research Center for Pharmaco-Toxicological Evaluation, “Victor Babes” University of Medicine and Pharmacy, 2 Eftimie Murgu Square, 300041 Timisoara, Romania; 7 Louvain Drug Research Institute, Université Catholique de Louvain, Avenue Emmanuel Mounier 73, 1200 Brussels, Belgium; 8 Faculty of Agriculture, University of Life Sciences “King Mihai I” from Timisoara, Calea Aradului 119, 300645 Timisoara, Romania; 9 Ineu City Hospital, 2 Republicii Street, 315300 Ineu, Romania

**Keywords:** multidrug-resistant strains, nosocomial infections, vancomycin-resistant *Enterococcus faecium*, clarithromycin-resistant *Helicobacter pylori*, fluoroquinolone-resistant *Campylobacter* spp., cephalosporin-resistant, fluoroquinolone-resistant *Neisseria gonorrhoeae*, fluoroquinolone-resistant *Salmonellae*, methicillin-resistant, vancomycin-resistant *Staphylococcus aureus*

## Abstract

Combating antimicrobial resistance (AMR) is among the 10 global health issues identified by the World Health Organization (WHO) in 2021. While AMR is a naturally occurring process, the inappropriate use of antibiotics in different settings and legislative gaps has led to its rapid progression. As a result, AMR has grown into a serious global menace that impacts not only humans but also animals and, ultimately, the entire environment. Thus, effective prophylactic measures, as well as more potent and non-toxic antimicrobial agents, are pressingly needed. The antimicrobial activity of essential oils (EOs) is supported by consistent research in the field. Although EOs have been used for centuries, they are newcomers when it comes to managing infections in clinical settings; it is mainly because methodological settings are largely non-overlapping and there are insufficient data regarding EOs’ in vivo activity and toxicity. This review considers the concept of AMR and its main determinants, the modality by which the issue has been globally addressed and the potential of EOs as alternative or auxiliary therapy. The focus is shifted towards the pathogenesis, mechanism of resistance and activity of several EOs against the six high priority pathogens listed by WHO in 2017, for which new therapeutic solutions are pressingly required.

## 1. Introduction

### 1.1. Antibiotics and Antimicrobial Resistance

Antibiotics (ABs) are common agents used in healthcare to treat and prevent potentially fatal bacterial infections [1]. Their introduction into clinical practice was arguably the greatest discovery of modern medicine in the twentieth century [2]. Yet, ABs have been around for millennia, as many of them are compounds synthetized by microorganisms to protect themselves and dominate different habitats [3]. While humans started exploiting the power of ABs as early as 1550 BC, it was not until circa 100 years ago that we managed to understand, synthesize and purify ABs, salvarsan and penicillin being the promoters of the AB era [1]. Mishandling ABs has resulted in the rapid expansion of antimicrobial resistance (AMR). Even though AMR is a naturally occurring process, it was first identified 50 years ago, when *Staphylococcus aureus* began to develop penicillin resistance [4]. AMR is commonly associated with the presence of AB-resistant genes (ARGs) in the bacterial genome [5]. Given that bacteria are able to pass on ARGs through vertical or horizontal gene transfer [6], there is clear evidence that prolonged exposure to ABs can easily turn non-resistant bacteria into resistant ones [7]. 

### 1.2. Main Determinants of AMR

Given that AMR is linked to the emergence of multidrug resistant (MDR) and extensive drug resistant (XDR) pathogens [8], it represents a serious global threat of growing concern that affects humans, animals and the environment [9]. According to the World Health Organization (WHO), AMR is annually responsible for the deaths of at least 700,000 people worldwide and the death toll could reach 10 million by 2050 [10]. There are several reasons why AMR occurs: (1) inappropriate prescribing of ABs; (2) dispensing ABs without a prescription; (3) poor AB regulations and lack of surveillance of resistance development; (4) excessive use of ABs in food-producing animals; (5) limited decontamination of wastewater; and (6) lack of research on new ABs [8,11]. 

Initial, therapy errors such as misdiagnosing the infection aetiology, choosing an incorrect dosage, overly extending the duration of treatment, overlooking the recommended guidelines regarding first line ABs or prescribing ABs without a clear clinical indication all raise the risk of developing AMR [11,12]. These mistakes might be due to gaps in knowledge; fear of complications from infections; concerns of not meeting perceived patient expectation; financial benefits or incentives, and misleading advertising from the industry [13]. Poor regulations in AB use contribute to medication abuse and the occurrence of AMR. Dispensing ABs without a medical prescription is associated with inappropriate drug choice, wrong dosage, shorter treatment course and increased risk of adverse drug reactions [14]. Unregulated overuse of ABs in hospitals has increased the rates of resistance in nosocomial infections and thus cross-transmission [8]. Confronting MDR bacteria has led many hospitals to introduce antimicrobial stewardship programmes to monitor antimicrobial use and identify ways to reduce development and transmission of AMR [15]. However, just reducing the consumption of ABs and not working on socio-economic factors will not have the desired impact on the prevalence of AMR [16].

Antibiotic use in the animal industry has been found to be a key element in the development of AMR. Given that ABs might stimulate the intestinal synthesis of vitamins and lower the competition for nutrients between host and bacteria, sub-lethal doses of ABs are commonly used as growth promoters for food-producing animals [17]. In developed countries, 60–80% of ABs are also given to animals, especially poultry, pigs and cattle [18], with penicillins, tetracyclines and sulphonamides having the highest AMR rates [19]. In addition, the use of ABs as preservatives in meat [20] or heavy metals (such as copper and zinc) as growth promoters led to a massive increase in AMR [17]. Moreover, ABs are able to reach the environment via human and animal excretions, improper disposal of unused drugs and waste streams from the production line [21]. If micropollutants—including ABs and microorganisms—are not successfully removed, they represent an important source of soil and water contamination with ARGs [22]. 

Researchers are unable to keep the pace of finding new ABs in the face of emerging MDR strains. Starting with the discovery of penicillin, many ABs discoveries were serendipitous, made by empirical screening [23]. Nevertheless, despite technological progress, the last 25 years have not seen a breakthrough in the development of novel antibacterial drug classes [24] and there is a critical need for drugs targeting Gram-negative ESKAPE pathogens [25].

### 1.3. How AMR Is Addressed Globally and at European Level

In 2015, WHO developed an action plan to combat AMR which includes, among other objectives, the reduction of AB use in humans and animals [26], followed in 2017 by the publication of a list of bacteria that urgently need the development of new ABs, dividing them into three classes based on their healthcare burden. The following bacteria were classified as having critical priority (priority 1): *Acinetobacter baumannii*, *Pseudomonas aeruginosa* and *Enterobacteriaceae*. High priority pathogens (HPPs) were categorized as priority 2: *Enterococcus faecium*, *Staphylococcus aureus*, *Helicobacter pylori*, *Campylobacter* spp., *Salmonellae* spp. and *Neisseria gonorrhoeae* [27]. In May 2022, WHO issued a review of ABs in development worldwide. Since their first analysis in 2017, 12 new ABs have been approved; however, most were derivatives of existing classes, where resistance mechanisms have been established. At the moment, 27 ABs targeting WHO priority pathogens are in clinical development with 13 having confirmed activity against at least one of the critical Gram-negative bacteria [28]. The incoming United Nations General Assembly in 2024 aims to identify clear and practical steps to address AMR [29].

The global AMR situation is briefly presented by One Health Trust’s Resistance Map [30]. However, this database only covers the resistance of a few HPPs and some ABs (Table 1), whereas the reality might be more severe. On the other hand, the AMR picture in Europe is presented by the Surveillance Atlas of Infectious Diseases [31,32]. Here, although the situation varies broadly from country to country, the highest prevalence seems to be in the south-eastern region (Appendix A, Table A1). Given that both the European and the global databases lack reports from several countries, the real magnitude of AMR is difficult to grasp.

### 1.4. Essential Oils as Potential Tools against AMR

Essential oils (EOs), also called volatile oils, are complex mixtures of tens of lipophilic, volatile compounds, at different concentrations [33]. Generally, two or three major components are present in rather high concentrations (20–70%), while other compounds are present in trace amounts [34]. The main constituents of EOs are terpenes—compounds derived from isoprene units that often have several chemical functionalities, such as alcohol, phenol, aldehyde, ketone, ether and hydrocarbon groups [35,36]. EOs represent secondary metabolites produced by aromatic plants as a protective mechanism against predators, microorganisms or austere weather conditions [37]. Many parts of the plant are able to produce EOs, which can be then extracted using methods such as solvent extraction (solvent, subcritical water, supercritical CO_2_), distillation (hydrodistillation, steam distillation, hydrodiffusion), solvent-free microwave extraction and combined methods [38]. The applications of EOs range from aromatherapy and perfume production to food industry and animal nutrition [39].

In medical practice, EOs have been reported to have antimicrobial, antioxidant, anti-inflammatory, analgesic, antiemetic and cancer chemo-protective activities [40,41]. Some EOs also exhibit cytotoxic (against bacteria, viruses, fungi, protozoa, parasites and mites), allelopathic and insect repellent and insecticidal activities, thus they could be exploited as alternative strategies in a variety of industries [40]. Certain EOs are effective against pathogens of public health interest. In a previous review paper, we have presented that several EOs exhibit in vitro antimicrobial activity against the WHO priority 1 list of pathogens [42]. Notably, it is generally considered that EOs are more active against Gram-positive than Gram-negative bacteria [40]. Interestingly, unlike classic ABs, EOs exhibit good activity against pathogenic bacteria, while showing a lower effect on beneficial bacteria in the gut, such as *Lactobacillus* or *Bifidobacterium* [43]. Thanks to developments in pharmaceutical formulations, EOs can be loaded in carriers such as nanoparticles which significantly improve their bioavailability and stability [44]. However, the general population can also benefit from the effects of antimicrobial EOs through the use of spices [45].

The chemical functionalities responsible for antimicrobial activity are generally phenols and aldehydes, while a high proportion of esters, ketones and terpene hydrocarbons result in weak or no effect [46]. Thanks to their hydrophobicity, EOs can inhibit bacterial growth by degrading membrane proteins and increasing cell permeability [46,47]. They can interfere with the expression of genes encoding efflux pumps (tetA, tetK, pmrA, norA, blaTEM, blaOXA-23) in a variety of microorganisms [48]. Proton pumps can also be affected by EOs, resulting in reduced membrane potential and ATP depletion [49]. Moreover, EOs can hinder biofilm formation and disrupt quorum sensing. Thus, they alter cell-to-cell communication and interfere with gene expression regulation—crucial adaptive measurement in hostile environments [50].

The two most common in vitro techniques used to assess the antimicrobial activity of EOs are the agar diffusion method (paper disc or well) and the dilution method (agar or liquid broth) [51]. The agar diffusion methods are one of the most convenient techniques, in terms of price and methodology. In the agar well diffusion method, an agar plate is inoculated with a pathogenic microorganism through the spreading plate approach (an exact volume of the microbial solution is spread over the surface of agar, through a glass diffuser). A well or hole is aseptically made with a sterile cork borer of diameter 6–8mm, flooded with the tested solution (e.g., extract) and incubated at optimal temperature and conditions. The tested solution will diffuse progressively through the agar medium, thus inhibiting the microorganism growth. Later, the diameter of the inhibition zone will be measured. In the agar disc diffusion method, a filter paper disc containing the test solution is placed on the agar medium and then inoculated with the tested strains [51]. Overall, the agar diffusion techniques make it possible to easily test several extracts/substances against various microorganisms, although they are unable to highlight the minimum inhibitory concentration (MIC) or the ability of a substance/extract to inhibit or kill a microorganism [51].

In the dilution method a series of successive dilutions of concentrated solutions of microbial strains are prepared to accurately count the viable cells within a culture (bacterial, fungus or viruses). Each diluted sample is added to a liquefied agar medium, poured into a petri dish and solidifies, holding the microorganisms within its matrix. It is possible to count the microorganisms with precision as they disperse across the agar plate. This method is used to determine the MIC of a substance, as well as its ability to kill or inhibit the development of the tested strains. Moreover, it is the reference for antimicrobial susceptibility testing [51].

Bacterial resistance is generally defined using the minimum inhibitory concentration (MIC) and minimum bactericidal concentration (MBC) of the selected AB. However, the reported values for MIC and MBC are extremely divergent, possibly as a result of the wide diversity of bacterial strains, methodological dissimilarities and study design variations [52]. Notably, a number of variables related to the variability of the extract (the part of plant used, extraction method, etc.) also have an impact on EOs’ antimicrobial activity [53,54].

The aim of this review is to investigate the current level of knowledge regarding the six WHO HPPs: vancomycin-resistant *E. faecium* (VREF); clarithromycin-resistant *H. pylori* (CRHP); fluoroquinolone-resistant *Campylobacter* spp. (FRC); cephalosporin-resistant, fluoroquinolone-resistant *N. gonorrhoeae* (CRNG, FRNG); fluoroquinolone-resistant *Salmonellae* spp. (FRS); and methicillin-resistant, vancomycin-intermediate and -resistant *S. aureus* (MRSA, VISA, VRSA). To our knowledge, there have not been any recent publications that systematically present the antimicrobial activity of EOs against WHO priority 2 pathogens. Thus, our two main objectives are to present (1) HPPs’ key resistance mechanisms and (2) the latest EOs that exert antimicrobial activity on the aforementioned pathogens, with an emphasis on the AB-resistant strains.

## 2. Methods

### 2.1. Review’s Objective and Design

The aim of this review was to investigate the current level of knowledge regarding the six WHO HPPs (priority 2 list). This study was designed as a follow-up of a previous paper that addressed WHO critical priority pathogens (priority 1 list) [42]. Each of the six bacteria were discussed in terms of: pathogenesis, mechanism of AMR (with an emphasis on ABs indicated by WHO as increasingly inefficacious) and EOs as potential treatment candidates (with an emphasis on EOs that are effective against resistant strains). A working team was set up with members having experience in the area of interest: microbiology, clinical laboratory, clinical pharmacy, botany and pharmacognosy.

### 2.2. Literature Search Strategy

**Figure 1:**
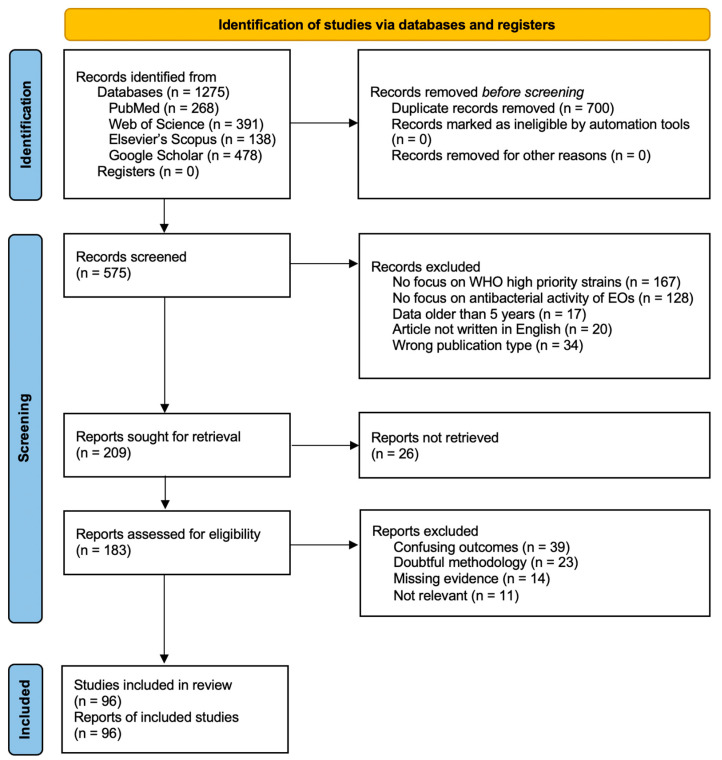
PRISMA 2020 flow diagram for new systematic reviews which included searches of databases and registers only: following database identification, duplicates were removed and the remaining records were screened for meeting the inclusion criteria. Retrieval of the full text was not possible for all publications. Before extracting the data from the studies, a final assessment was carried out to eliminate publications whose findings were ambiguous, according to the exclusion criteria.

A flow diagram of screened records is provided in Figure 1. We conducted an independent search for each bacterium on three electronic databases (PubMED, Web of Science, Elsevier’s Scopus) and one web search engine (Google Scholar) from April 2022 to September 2022. The primary keyword combination used to perform the search was the following: WHO critical pathogen strain AND AB resistance AND essential oils (e.g., *Enterococcus faecium* AND vancomycin-resistant AND essential oils). Searches were conducted separately in each database and, after removing the duplicates, the records were exported to the citation software. Articles included in the study were those that: (1) focused on WHO priority 2 strains (primarily aiming for MDR strains); (2) investigated the activity of EOs against pathogens; (3) were published in the last 5 years; and (4) were written in English. The articles that failed to meet the inclusion criteria were excluded from the study, as well as the ones that fell under one of the following categories: book chapter, abstract, short communication, technical note, letter. Publications that appeared to be methodologically flawed and provided insufficient details or confusing outcomes were also dismissed.

### 2.3. Data Extraction

Pertinent articles were closely evaluated by reviewers and the following data were extracted: (1) study team and year of publication; (2) bacterial strain under investigation (including its resistance, if mentioned); (3) EOs/pure phytocompound proposed as having antimicrobial activity; method(s) of assessing the antimicrobial activity; and (4) main results. Any disagreements or queries were settled by a researcher with expertise in microbiology (D.M.).

## 3. Results and Discussion

### 3.1. Enterococcus Faecium, Vancomycin-Resistant

*Enterococcus* spp. are Gram-positive bacteria that are part of human faecal microbiota, as commensals of the intestinal tract [52]. However, some strains, particularly *Enterococcus faecalis* and *Enterococcus faecium*, may cause nosocomial outbreaks and opportunistic infections in hospitalized patients [53]. While most enterococci infections are caused by *E. faecalis* (nearly 90%), *E. faecium* has a higher rate of AB resistance, especially in case of bloodstream infections [54]. Plasmid transfer and homologous recombination, mediated by insertion sequence elements, are two important mechanisms through which *E. faecium* manages to escape effective therapy [55]. A series of genes (*ace*, *acm*, *scm* and *ecb*) code for a subset of adhesion factors that mediate the pathogen’s initial attachment. Aggregation substances, such as Enterococcal surface protein, favour *E. faecium*’s ability to form biofilms. Moreover, exoenzymes, including gelatinase, hyaluronidase and cytolysin, are secreted externally and can damage host cells by triggering inflammation [56].

Until recently, vancomycin was considered the gold standard in the therapy of beta-lactam resistant Gram-positive cocci. However, AMR is rising: in the United States, 30% of enterococci are vancomycin-resistant [57], while in some European countries the proportion of VREF is even higher (Table 1). VREF carried by patients is commonly shared within the hospital environment, as it may persist despite standard cleaning; thus, patients can acquire VREF if they are admitted to a room previously occupied by a VREF-positive patient [58]. VREF can be detected by identifying at least one of nine resistance genes—*vanA*, *B*, *C*, *D*, *E*, *G*, *L*, *M* and *N*—present in the mobile genetic elements (*vanA*, *vanB*) or located on the chromosome (*vanC*) [59]. The most predominant genotypes are *vanA* and *vanB*. *vanA E. faecium* is highly resistant to vancomycin and teicoplanin, while *vanB E. faecium* shows high resistance to vancomycin and susceptibility to teicoplanin [60]. Although linezolid has been considered a good alternative in treating VREF infections, resistance to this drug is gradually increasing, with G2576 T mutation within the 23S rDNA being one of the major resistance mechanisms [57]. Teicoplanin or last-line ABs such as daptomycin, tigecycline or quinupristin/dalfopristin remain an option for some VREF infections, but resistance has been reported [61,62].

There are several EOs that have shown activity against VREF (Appendix B, Table A2). Saki et al. investigated the antibacterial effects of cinnamon bark (*Cinnamomum zeylanicum*) EO on XDR isolates and determined that VREF was sensitive to this EO [63]. Five other EOs, namely bitter orange (*Citrus aurantium v. amara*), lemon (*Citrus × limon*), blue gum eucalyptus (*Eucalyptus globulus*), tea tree (*Melaleuca alternifolia*) and Mediterranean cypress (*Cupressus sempervirens*) were evaluated by Iseppi and collaborators. They observed that *M. alternifolia* EO was the most effective EO and *C. aurantium* the least effective EO, while the other three EOs displayed antibacterial activity against all strains, to a certain extent. What is more, EO–EO and EO–AB associations showed a synergistic antimicrobial activity in most tests and were even effective against biofilm formation [64]. Synergistic interactions were also investigated by Owen et al. in their study on oregano (*Origanum compactum*), rosewood (*Aniba roseadora*) and cumin (*Cuminum cymimum*) EOs. They found out that the combination of carvacrol and cuminaldehyde could re-establish susceptibility to vancomycin in VREF, resulting in bactericidal activity [65]. Their previous studies also indicated that these particular EOs exhibited zones of inhibition against VSEf and VREF in the Kirby–Bauer disc diffusion method, with minimal inhibitory concentrations (MICs) ranging from 0.29 to 37.20 mg/mL [66].

When looking into the effect of five EOs on VREF isolated from wastewater treatment plants, clinical samples and reference strains, Sakkas et al. discovered that origanum (*Thymus capitatus*), thyme (*Thymus vulgaris*) and tea tree (*Melaleuca alternifolia*) EOs were effective against the pathogenic strains [67]. Di Vito et al. compared the antimicrobial activity of EOs and hydrolates extracted from lavender (*Lavandula angustifolia* and *Lavandula intermedia*), origanum (*Origanum hirtum*), winter savory (*Satureja montana*), scarlet beebalm/bergamot (*Monarda didyma*) and beebalm/wild bergamot (*Monarda fistulosa*). They determined that the antimicrobial activity of hydrolates is milder than that of the corresponding EOs, with higher MICs. In contrast to hydrolates, which must be used at concentrations of 25–50% *v*/*v* to achieve the same antimicrobial activity, EOs are active at lower concentrations: 50% of the strains are susceptible at concentrations of 0.125–2% *v*/*v* [68].

Further data from these studies are presented in Appendix B, Table A2.

### 3.2. Helicobacter Pylori, Clarithromycin-Resistant

*Helicobacter pylori* are Gram-negative bacteria that colonize the stomach and duodenum, and might be a key contributor in diseases such as chronic gastritis, peptic ulcer, gastric adenocarcinoma, mucosa-associated lymphoid tissue lymphoma and iron deficiency anaemia [69]. While the most common sources of *H. pylori* contamination are water, environment and animals [70], it appears that a plant-based diet is associated with a lower prevalence in adults; in contrast, higher consumption of fried food and well water are considered risk factors for infection [71]. Overall, prevalence of *H. pylori* infection seems to be declining globally, with Oceania having the lowest prevalence rates (24.4%); however, in some parts of the world, the prevalence remains quite stable and reaches a rate of 79.1% in Africa [72]. *H. pylori* can cause lifelong infection without eradication, with recurrence appearing either by recrudescence or reinfection [73]. It has been established that *H. pylori* gastritis should be considered an infectious disease regardless of the absence of symptoms, complications or resultant illnesses [74]. Several specific virulence factors can lead to a more severe outcome: cytotoxin-associated gene A (*CagA*, especially EPIYA-D and EPIYA-C) and type IV secretion system (*CagL* polymorphism), the genotypes of vacuolating cytotoxin A (*vacA*, s1/i1/m1 types), and blood group antigen binding adhesin (BabA, low-producer or chimeric with BabB) [75].

The classical triple-therapy regimen against *H. pylori* infection includes a proton-pump inhibitor (PPI), amoxicillin and clarithromycin, taken together for 7–14 days [76]. The addition of nitroimidazole (metronidazole or tinidazole) adds benefits to the regimen, requiring fewer doses of ABs and being more effective [77]. Due to its ability to inhibit bacterial protein synthesis, clarithromycin has been the mainstay of therapy in *H. pylori* infections; the AB has a good mucosal diffusion, low MIC and a minimal impact on gastric acidity [78]. However, vertically transmitted point mutations in the peptidyl transferase loop of the V domain of 23S rRNA gene are leading to the emergence of CRHP strains [79]. In developed countries, 90% of CRHP appear as a result of mutations in two specific adjacent nucleotide positions—A2142G and A2143—which are the most prevalent and well-documented point mutations in this pathogen [80]. Efflux pumps and outer membrane proteins are also involved in clarithromycin-resistance. Notably, PPIs are structurally similar to efflux pump inhibitors, such as Phe-Arg-β-naphthylamide—a molecule able to decrease the MIC of ABs. As CRHP continues to escape efficient therapy, new drugs have been developed, such as vonoprazan—a novel potassium-competitive acid blocker that strongly inhibits H+/K+ ATPase-mediated gastric acid secretion [81]. In May 2022, The US Food and Drug Administration has approved two vonoprazan-based treatments, both superior to PPI-based triple or dual therapy [82].

Elkousy et al. demonstrated that both marjoram (*Origanum majorana*) and mandarin (*Citrus reticulata*) EOs exhibited antimicrobial activity against *H. pylori*. The authors stipulated that, thanks to its high content of oxygenated compounds (trans-sabinene hydrate, terpinen-4-ol, linalyl acetate, caryophyllene oxide and a-terpineol), marjoram EO displayed a lower MIC than the other EO. Additionally, the combination of the two EOs had a synergistic effect against *H. pylori*, with a lower MIC, equal to clarithromycin’s MIC [83] (Appendix B, Table A3). A study that investigated the effectiveness of four *Piper* spp. EOs against *H. pylori* determined that long pepper (*Piper longum*) EO recorded the same MIC as clarithromycin, followed by white pepper EO, tailed pepper and then black pepper. This study shows that, although the EOs came from plants in the same genus, individual components in each volatile oil led to variation in MIC values [84]. The same is true for different *Pinus* species, as Gad et al. determined that, among four pine EOs, *P. pinea* EO exhibited the highest anti-*H. pylori* activity, with a MIC comparable to that of clarithromycin [85]. In a study by Mariem et al., 54.54% of gastric biopsy *H. pylori* isolates showed resistance to at least one of the five tested ABs (erythromycin, clarithromycin, ciprofloxacin, levofloxacin or metronidazole), while mastic tree (*Pistacia lentiscus*) EO showed anti-*H. pylori* activity against all tested strains [86]. Several other EOs (wild thyme [87], cinnamon [88], cedarwood, oregano [89]) exhibited strong antimicrobial activity against *H. pylori* strains, while other EOs (common sage, lemon balm, English lavender [87], clove, thyme, rosemary [88], guabiraba [90]) showed good to mild antimicrobial effects. Various active compounds/EOs, such as geraniol [91], *α*-pinene (from *P. atlantica*) [92] or *β*-caryophyllene [93] are increasingly being tested on animal models, uncovering their anti-*H. pylori* activity (Appendix B, Table A3). However, data regarding EOs’ activity against CRHP are scarce.

### 3.3. Campylobacter spp., Fluoroquinolone-Resistant

*Campylobacter* spp. includes several commensal Gram-negative species, but also pathogenic strains of *Campylobacter jejuni* and *Campylobacter coli* [94]. *C. jejuni* alone is the leading cause of bacterial gastroenteritis in humans, surpassing *Escherichia coli*, *Shigella* spp. and *Salmonella* [95]. The main sources of contamination are raw or undercooked meat, unpasteurized milk or dairy products, contaminated vegetables, natural mineral water, shellfish and flies [96]. In developing countries, *Campylobacter* infection is generally limited to children and the clinical symptoms are usually indistinguishable from those caused by other enteric pathogens [97]. In some instances—usually in immunocompromised, pregnant and elderly patients—*Campylobacter* can trigger extraintestinal manifestations, including abscesses, meningitis, endocarditis and bacteraemia [98]. Moreover, a number of patients develop chronic sequelae, such as irritable bowel syndrome, reactive arthritis, Reiter’s Syndrome, Guillain–Barré Syndrome, Crohn’s disease and ulcerative colitis [99]. The main virulence factors of *Campylobacter* spp. are their ability to adhere and colonize (*cadF*, *racR*, *virB11*, *pldA* and *dnaJ* genes), invade intestinal epithelial cells (*ciaB* and *ceuE* genes) and produce toxins (*cdtA*, *cdtB*, *cdtC* genes) [100].

While self-limiting intestinal manifestations are treated using replacement of fluids and electrolytes, severe extraintestinal infections need treatment with the ABs of choice (macrolides and fluoroquinolones [101]) or with alternative solutions (tetracyclines and aminoglycosides), for which all have reported AMR [102]. Fluoroquinolones act by inhibiting DNA gyrase (GyrA and GyrB subunits) and topoisomerase IV (ParC and ParE subunits)—two enzymes crucial for bacterial survival, involved in DNA replication and repair, transcription and recombination [103]. Point mutations (T86I, T86K, A70T and D90N) in the quinolone-resistance-determining region (QRDR) of DNA gyrase’s GyrA subunit led to the emergence of FRC [104]. Moreover, drug efflux pump CmeABC works synergistically with the gyrA mutation, leading to the appearance of FRC; however, in the absence of the gyrA gene mutation, CmeABC over-expression does not correlate with ciprofloxacin resistance [105]. Nevertheless, fluoroquinolone resistance cannot be attributed to mutations in *parC* and *parE* genes (since Campylobacter lacks these particular genes); similarly, it appears that mutations in the *gyrB* gene are not related to the emergence of FRC [106].

Gahamanyi et al. evaluated the susceptibility of *C. jejuni* and *C. coli* to various natural products (plant extracts, EOs and active compounds) and frontline ABs. They determined that, among the tested products, cinnamon (*Cinnamomum cassia*) EO and its main compound (E)-Cinnamaldehyde, clove (*Syzygium aromaticum*) EO and its main compound eugenol, and baicalein had the lowest MIC and MBC values (25–100 µg/mL) [107] (Appendix B, Table A4). Duarte et al. tested the activity of coriander (*Coriandrum sativum*) EO and its major compound linalool against the two above-mentioned strains; once again both products showed comparable activities: inhibition of biofilm formation, reduction in quorum-sensing and inhibition of the microbial growth [108]. The anti-*C. jejuni* activity of clove EO and eugenol was supported by another study showing that the products perturb the expression of virulence factors, alter the morphology and induce oxidative stress in *C. jejuni* [109]. Ahmed et al. confirmed that clove and cinnamon EOs were effective against *C. jejuni* and *S. aureus*, but garlic EO did not share the same efficacy [110]. El Baaboua et al. determined that MDR *Campylobacter* spp. were sensitive to oregano (*Origanum compactum*), mint (*Mentha pulegium*) and lavender (*Lavandula stoechas*) EOs, which interfered with the microbial ability to form biofilms. Lavender and oregano acted synergistically with tetracycline or ampicillin, reducing the effective doses of EOs, tetracycline and ampicillin [111]. One interesting study investigated the potential of thyme (*Thymus vulgaris*) EO/gelatin nanofibers to inhibit *C. jejuni* growth in chicken. Food packaging containing thyme EO *β*-cyclodextrin *ε*-polylysine nanoparticles as antibacterial agents readily damaged *C. jejuni* cell membranes and reduced the microbial population [112]. Another noteworthy approach was that of Lin et al. who prepared chrysanthemum EO–chitosan–pectin triple-layer liposomes and determined that the product exhibited high anti-*C. jejuni* activity [113]. Lastly, even though some active compounds present promising in vitro results, further in vivo studies fail to reach the same outcomes. For instance, although (-)-*α*-pinene was able to reduce ciprofloxacin’s MICs (when used in combination), it did not manage to impede fluoroquinolone resistance development when added to enrofloxacin in broiler chickens [114] (Appendix B, Table A4).

### 3.4. Neisseria gonorrhoeae, Cephalosporin-Resistant, Fluoroquinolone-Resistant

*Neisseria gonorrhoeae* is a Gram-negative diplococcus and represents the etiological agent of gonorrhoea, the second most common sexually transmitted infection that affects both men and women [115]. According to WHO, *N. gonorrhoeae* causes significant morbidity and economic costs around the world, with 82.4 million new cases of gonorrhoea among adults and adolescents each year [116]. While the majority of gonorrhoea cases are asymptomatic, untreated infections may lead to serious complications such as endometritis, salpingitis, sterility, chronic pelvic pain, ectopic pregnancy, neonatal infections and increased risk of acquiring HIV [117]. Moreover, there are cases when the pathogen disseminates, causing skin, joint or tendon infection and, rarely, endocarditis or meningitis [118]. The primary step in *N. gonorrhoeae* pathogenesis is the bacterial adherence to the epithelium of the mucosa, mediated through surface structures: type IV pili, opacity (Opa) proteins, LOS and PorB—the major outer membrane protein porin [119]. After adhering, the pathogen replicates, forming microcolonies and biofilms, and sometimes even invades epithelial cells by transcytosis [120].

For many years, gonorrhoea was considered relatively easy to treat in monotherapy; however, due to AB overuse, AMR has emerged for most classes (sulphonamides, penicillins, tetracyclines, macrolides, fluoroquinolones and early-generation cephalosporins) [121]. Today, the most recommended gonorrhoea treatment is dual therapy: a single dose of a third-generation cephalosporin (250–500 mg intramuscular ceftriaxone or 400 mg peroral cefixime) in combination with azithromycin (1–2 g peroral) [122]. Concerningly, in 2018, ceftriaxone-resistant and azithromycin-resistant *N. gonorrhoeae* strains have been isolated [123]. Cephalosporin resistance is caused by mutations in various chromosomal regions that encode for important microbial proteins such as the transpeptidase domain of the PBP2 protein (*penA* gene), porin B subunit (*porB1b* gene) and PBP1 protein (*ponA* gene) [124], as well as overexpression of MtrCDE membrane pump proteins [125]. Fluoroquinolone resistance is acquired through mutations in QRDR: a single amino acid change in the GyrA subunit (positions 91 or 95) leads to intermediary resistance level, while three or more changes in the GyrA subunit (positions 91, 95 and 102), ParC (position 87 and 91) and/or Par E (position 439) proteins may lead to even higher MICs [126]. There is a pressing need to develop new diagnostic strategies and novel antimicrobials in order to preserve ceftriaxone, as it is the last empirical first-line monotherapy for gonorrhoea [127]. In 2021, WHO issued a general protocol called the Enhanced Gonococcal Antimicrobial Surveillance Programme, which aims to strengthen the quality, comparability and timeliness of gonococcal AMR data across multiple countries [128]. As the global burden rises, new collaborators join this programme to combat AMR in gonorrhoea [129].

Propolis extract has been shown to exhibit antimicrobial activity against ciprofloxacin-sensitive and ciprofloxacin-resistant *N. gonorrhoeae* strains, in a study by Vică et al.; depending on the harvesting region, the extracts presented with various inhibition zone diameters and MICs [130] (Appendix B, Table A5). Umaru et al. studied the effect of *Molineria capitulate* fruit EO on various pathogens and showed that both *M. capitulate* EO and its major component, myrcene, displayed antimicrobial activity against *N. gonorrhoeae* strains [131]. Soliman et al. investigated the antimicrobial properties of two guava (*Psidium* spp.) EOs and determined that both EOs showed good antibacterial effects, but *Psidium cattleianum* displayed preferential activity against *N. gonorrhoeae* [132]. Other publications showed that *Eclipta alba* EO is highly effective against *N. gonorrhoeae* strains [133], while *Ferula tingitana* EO displays a more modest antibacterial activity [134] (Appendix B, Table A5).

### 3.5. Salmonellae, Fluoroquinolone-Resistant

*Salmonella* spp. is a genus of facultative anaerobe Gram-negative bacilli having peritrichous flagella [135]. The genus *Salmonella* belongs to the family Enterobacteriaceae and includes two main species: *Salmonella enterica* and *Salmonella bongori* [136]. Around 99% of the *Salmonella* strains that cause infection in humans or other mammals belong to the *S. enterica* strains [137]. *S. enterica* is further subdivided into six-subspecies, serotypes, serogroups and serovars, according to the expression of somatic lipopolysaccharide O and flagellar H antigens [138]. The three major diseases caused by *Salmonella* in humans are: non-invasive non-typhoidal salmonellosis (niNTS), invasive non-typhoidal salmonellosis (iNTS) and typhoid fever [139]. Additionally, *Salmonella* serotypes can asymptomatically colonize humans’ gallbladders, thus making them chronic carriers and potential disseminators [136].

Non-typhoidal salmonellosis (NTS) is among the most prevalent global cause of foodborne illnesses [140] and includes infections caused by all *Salmonella* spp., with the exception of the distinct typhoidal serotypes: Typhi and Paratyphi A-C [139]. While niNTS may have a variety of clinical manifestations, the most common is gastroenteritis and is usually self-limiting [140]. Given that both humans and animals are potential hosts for niNTS [141], the infection is mainly transmitted via the consumption of animal products, but also unpasteurized dairy products, seafood and fruits [140]. The incidence of gastroenteritis due to niNTS peaks in the developing world, but it is also of considerable importance in developed countries; for instance, in the European Union (EU), salmonellosis is the second-most reported gastrointestinal infection in humans after campylobacteriosis [140]. When non-typhoidal *Salmonella* spp. (*S. typhimurium* and *S. enteritidis*) go beyond the gastrointestinal tract and invade normally sterile sites causing bacteraemia, iNTS occurs [142]. Contrary to niNTS, iNTS typically manifests as a febrile systemic illness (where diarrhoea is often absent) and lower respiratory tract disorders, due to co-infections with *Mycobacterium tuberculosis* and *Streptococcus pneumoniae* [139]. The global burden of iNTS is estimated by Ao et al., who evaluated 3.4 million cases of iNTS and approximated the annual death toll to around 700,000 [143]. In a similar study that excluded patients with HIV-associated iNTS, it was established that more than 60,000 deaths occurred in 600,000 cases of iNTS [142]. The most severe *Salmonella* spp. infections, typhoid and paratyphoid enteric fevers, are caused by *Salmonella enterica* subspecies *enterica* serovars Typhi and Paratyphi A, B and C [137,141]. Contrary to NTS broad host specificity, *S. typhi* is found only in humans [139]. For reasons not fully understood, it is estimated that around 5% of infected individuals will fail to clear the infection within a year, and will instead progress to a chronic carrier state where the bacteria will primarily reside in the hepatobiliary tract and gallbladder [139]. These systemic diseases cause more than 200,000 deaths globally, sub-Saharan Africa and Asia accounting for around 46% and 32% of typhoid fever cases, respectively [137]. Worryingly from 1990 to 2010 annual mortality from typhoid fever has increased by 39% [139].

Antibiotic therapy is not needed for *Salmonella*-induced gastroenteritis while, for invasive *Salmonella* infections, ampicillin, chloramphenicol and trimethoprim-sulfamethoxazole are used as first-line treatment [136]. However [139], a large proportion of AB-resistant *Salmonella* are acquired through the consumption of contaminated food of animal origin [144]. Poultry are the main source of human salmonellosis. In order to move forward through the food chain, *Salmonella* must be resistant to different environmental stress conditions such as heat, desiccation, nutrient starvation or biocides, and *Salmonella* spp. use quorum sensing to control processes such as luminescence, sporulation, virulence or biofilm formation [135].

AMR can be achieved by mutations in different chromosomal loci that are part of a core set of genes, such as genomic islands and through exogenous resistance genes carried by mobile genetic elements that can diffuse horizontally [145]. *Salmonella* spp. mediate resistance to ABs by three major mechanisms: drug inactivation, protection of the AB target sites, and removal of ABs using efflux pumps or multidrug pumps. Firstly, the main mechanism, drug inactivation, is characterized by destruction of antimicrobial agents (quinolones, macrolides) through chemical modification using enzymes that catalyse reactions such as acetylation, phosphorylation and adenylation [144]. Secondly, *Salmonella* spp. can protect the target sites of ABs, which are typically either enzymes or other specific cell structures [144,146]. For instance, the plasmid-encoded quinolone resistance protein (Qnr) confers resistance to quinolones by acting as a DNA homolog that competes for the binding of DNA gyrase and topoisomerase IV [144,146]. Thirdly, *Salmonella* spp. can use relatively nonspecific efflux pumps (such as AcrAB-TolC) encoded by genes within mobile elements to reject fluoroquinolones, β-lactams and carbapenems [144,146].

Emerging MDR *Salmonella* spp. have changed the treatment regimen towards using second-line ABs such as fluoroquinolones (ciprofloxacin) and third-generation cephalosporins [136]. However, as fluoroquinolones are extensively exploited for animal production in several countries [147], WHO notifies that the prevalence of FRS is growing quickly [136]. Under normal conditions, quinolones enter bacteria through porins and then exert their bacteriostatic activity by binding to the gyrase/topoisomerase IV–DNA complex [144]. Resistance to nalidixic acid, driven by a single mutation within *gyrA*, is a precursor to resistance to all quinolones [148]. Additionally, the fluoroquinolone resistance in *Salmonella* is caused either by chromosomal mutations in the QRDRs of the *gyr* and *par* genes [149], or by the acquisition of several plasmid-mediated quinolone resistance (PMQR) genes [143]: (1) *qnr *(quinolone resistance proteins); (2) *aac(6′)-lb-cr* (aminoglycoside-modifying acetyltransferase); and (3) *oqxAB* and *qepA* [141,144]. Increased expression of the AcrAB-TolC multidrug efflux system was also shown to play an important role in the development of high-level fluoroquinolone resistance [149]. Generally, resistance to ciprofloxacin in enteric bacteria is acquired through *gyrA* mutations, while PMQR genes also induce low-level resistance. In contrast, in *Salmonella* spp., mutations in transferable PMQR gene *qnr* were observed in all ciprofloxacin-resistant isolates, whereas *gyrA* mutations were often found in isolates with reduced ciprofloxacin susceptibility but not in all ciprofloxacin-resistant isolates [150].

To our knowledge, there are no articles describing the antimicrobial action of EOs on FRS.

### 3.6. Staphylococcus aureus, Methicillin-Resistant, Vancomycin-Intermediate and -Resistant

*Staphylococcus aureus* is a ubiquitous Gram-positive, coagulase-positive, facultative anaerobe, nonmotile and spherical bacterium (grape-like cluster), possessing not a flagella but a capsule [151]. It can be present either as a commensal member of the microbiota (usually localized in the upper respiratory tract, gut or on the skin) or as an opportunistic pathogen [152]. *S. aureus* may cause mild to severe infections, depending on the localization of the infection (skin, soft tissues or blood) and patient’s characteristics (age—more aggressive in infants and older people; comorbidities—immunosuppression, diabetes, heart or renal diseases; and others factors—implantable medical devices, low social economic status or intravenous drug use) [153,154]. The disorders caused by *S. aureus* range from skin infections (pimples, impetigo, folliculitis, boils, cellulitis, carbuncles, scalded skin syndrome, abscesses) to more severe and life-threatening diseases, including pneumonia, osteomyelitis, endocarditis, meningitis, toxic shock syndrome, bacteremia or sepsis [151,155].

While most people who are colonized with *S. aureus* will not develop an invasive infection, *S. aureus* infections are overall extremely frequent and particularly problematic due to AB resistance and the ability to form biofilms [153]. In 1942, shortly after the introduction of penicillin into clinical practice, *S. aureus* started hydrolysing the beta-lactam ring and establishing penicillin resistance. Then, in the late 1950s, the appearance of the semi-synthetic beta-lactamase-resistant AB, methicillin, led to the emergence of the first MRSA strain only 2 years after its introduction [156] due to mutations in the gene encoding for penicillin-binding protein 2a or 2′ (PBP2a; PBP2′) (*mec*A) [152]. Nowadays, *S. aureus* remains one of the most common resistant pathogens worldwide [154,157]. According to the American Centers for Disease Control and Prevention, *S. aureus* has evolved into a major health-threatening pathogen, as invasive infections caused by MRSA have a high mortality rate and its remarkable ability of acquiring AB resistance against multiple drug classes significantly complicates the treatment [158]. Although the rates of AB resistance are widely variable depending on the country (relatively low AMR in Scandinavian countries and high AMR in Southern Europe, USA and China, due to differences in hygiene and surveillance measures), they are still on the rise in poorly developed countries (South America and some countries in Africa) [151,159].

The beginning of the 1960s was marked by the discovery of the SCC*mec* complex, a key factor that enabled *S. aureus* to acquire resistance to most of the beta-lactam ABs [151]. Currently 12 SCC*mec* complexes are known, and they are divided by (1) the group of cassette chromosome recombinase (ccr) complex and (2) the category of *mec* complex. With the exception of type XI SCC*mec* which contains homologue *mec*C, all SCC*mec* types include *mec*A, a component that encodes for PBP2a [160]. PBP2a is responsible for the transpeptidase action in the biosynthesis of peptidoglycan, in the presence of beta-lactam AB, inhibiting the function of PBP 1, 2, 3 and 4. While *mec*C is a variant of *mec*A which encodes for PBP2aLGA251 (named after MRSA strain LGA 251), *mec*B is a plasmid-developed methicillin resistant form with unclear mechanism of resistance [161,162]. *mec*A’s expression depends on regulators encoded by *mec*I*, mec*R1 and *mec*R2, and on regulators of gene expression, such as *bla*Z, *bla*I and *bla*RI [162]. The auxiliary *fem* genes seem to also have an important influence on the resistance phenotypes [163].

Over time *S. aureus* has also gained resistance to vancomycin, the AB used as a first-line treatment for the past six decades [164,165]. The feasible alternative options to vancomycin treatment include high doses of daptomycin co-administered with either gentamicin, rifampicin, linezolid, trimethoprim + sulfamethoxazole or beta-lactam. If response to daptomycin is inadequate, secondary options include monotherapy or co-administration with a number of possible ABs: quinupristin + dalfopristin, linezolid, telavancin or trimethoprim + sulfamethoxazole [166]. In 2002, VRSA strains were identified in the USA; it is thought that resistance was mediated by *van*A gene acquired from *E. faecalis* on the plasmid-borne transposon Tn1546 [167]. Given that VRSA has a preference for diabetic wounds where vancomycin-resistant enterococci reside, there is a clear opportunity for horizontal gene transfer of Tn1546 accommodating *van*A [168].

The study performed by Oo T et al. evaluated the antimicrobial efficacy of crude extract and EO obtained from nutmeg (*Myristica fragrans* Houtt.) on *S. aureus* efflux pump systems (chromosomal norA and mepA) involved in the resistance mechanism of MRSA. They found that elemicin, myristicin, methoxyeugenol and asarone can work as efflux pump inhibitors, thus potentiating the antimicrobial activity of classical ABs. They observed a synergistic activity of ciprofloxacin and the two nutmeg formulations, concluding that both the extract and the EO act as efflux pump inhibitors, while ciprofloxacin acts as an efflux system [169] (Appendix B, Table A6). Alharbi NS et al. highlighted the effect of two different concentrations of tailed pepper (*Piper cubeba* L.) EO against MRSA ATCC 43300. While the higher concentration induced serious microscopic deteriorations of the bacterial cell, the lower concentration had no observable microscopic effects; however, significant modifications within the cell wall were observed at a nanoscopic level. The authors concluded that the EO induced an antibacterial action on both methicillin- and oxacillin-resistant *S. aureus* strains through its action upon the cell wall and the cytoplasmic membrane [170] (Appendix B, Table A6).

Piasecki B et al. tested 19 EOs extracted from *Cymbopogon* spp. and determined that *C. flexuosus* (lemongrass) EO exhibited the highest antibacterial activity, while citronellol stood out as the most powerful active compound (from citronellol, geraniol and citral). Moreover, all tested EOs manifested antibiofilm properties, with a MBIC ranging from 1 to 4 mg/mL. Nonetheless, after 48 h of treatment at a maximum concentration, cardiotoxicity and shortened tail were observed in zebrafish, with *C. martini* var. *motia* showing the most toxic potential (about 20 times more toxic than *C. winterianus*) [171]. Merghni A et al. also showed that both blue gum (*Eucalyptus globulus* Labill.) EO and its main active compound, 1,8-cineole, present excellent antibiofilm properties and bacteriostatic effects, with the EO inducing a more potent effect on quorum sensing [172] (Appendix B, Table A6).

It is well known that Gram-negative bacteria are more resistant to ABs and toxins than Gram-positive bacteria, due to their perfected cell wall. Specifically, they have a multi-layered, complex cell wall, covered by a hydrophilic membrane abundant in lipopolysaccharides. Moreover, the periplasmic space of Gram-negative bacteria contains enzymes capable of degrading exogenous molecules, thus preventing the access of inhibitors. Several studies have also emphasized that there is a difference in the antibacterial activity of EOs in Gram-positive versus Gram-negative bacteria [173] (Appendix B, Table A6).

Predoi D et al. investigated the activity of EOs in hydroxyapatite, a calcium phosphate compound best known for its similarities with human hard tissues and its applicability in medicine (dental applications, bone regeneration). Their work was based on the premise that, by incorporating antibacterial agents in hydroxyapatite, the risks of postoperative infections would be reduced. Thus, they highlight the physio-chemical properties and antimicrobial activity of two nanocomposites of hydroxyapatite embedded with basil and lavender EOs, with the latter exhibiting the best antibacterial properties [174]. The team continued to look into the effectiveness of hydroxyapatite associations with EOs in further studies [175,176] (Appendix B, Table A6).

Interestingly, Mouwakeh A and collaborators postulated the fact that black caraway (*Nigella sativa* L.) EO and its active components (carvacrol and p-cymene) could be used as MRSA modifiers of resistance. In their study they hypothesized that the hydroxyl group in carvacrol and thymoquinone might play a key role in their antimicrobial activity [177].

De Moura et al. focused on evaluating the antioxidant, antibacterial and antibiofilm activity of nerolidol, an acyclic sesquiterpene present in many species (usually the trans isomer) such as wood oil, red oil, cabreuva oil and balm of Peru [178]. It is of valuable importance in the cosmetic industry (as a preservative agent to fixate perfumes) and pharmaceutical industry (as a stimulating agent to increase the concentration of active ingredients in transdermal formulations) [179]. Nerolidol has a broad spectrum of pharmacological/biological actions, including antineoplastic, anti-inflammatory, antinociceptive, larvicidal and leishmanicidal activity [179], and, recently, its implication for neurodegenerative disorders has been described [180]. De Moura and colleagues found that nerolidol is effective for both MSSA and MRSA at the same MIC of 2 mg/mL [178].

Two EOs of lemon verbena (*Aloysia citriodora* Palau) collected from different Palestinian regions were tested for their antimicrobial, antioxidant, cytotoxic and cyclooxygenase (COX) inhibitory effects by Jaradat N et al. Both EOs showed antimicrobial activity against MRSA, *P. vulgaris* and *C. albicans*, but the Baqa al-Gharbiyye EO manifested stronger antioxidant, cytotoxic and anti-cyclooxygenase activities, compared with the Umm al-Fahm EO [181] (Appendix B, Table A6).

The work of Adrian Man and collaborators investigated the antimicrobial effect of some well-known EOs in micellar and aqueous extracts. They assessed the antibacterial activity of oregano, lemon, thyme, myrtle and frankincense EOs against *S. aureus*, *E. faecalis*, *E. coli*, *K. pneumoniae* and *P. aeruginosa*, and determined that gram-positive bacteria (including MRSA) were more susceptible compared with *P. aeruginosa* which was found to be the most resistant. The authors highlighted that micellar suspensions of EOs (especially those containing high concentrations of terpenes and terpenoids—i.e., oregano, thyme, lemon EOs) can be introduced in new topical formulations to enhance the penetrability of EOs and, thus, their action [41] (Appendix B, Table A6).

The study of Kwiatkowski P. addressed the efficacy of several active compounds in EOs against mupirocin-susceptible and low-level mupirocin-resistant MRSA. Notably, mupirocin is an AB synthesized by *Pseudomonas fluorescens* and has medical applications. It is used on nasal mucosa as an ointment in order to decolonize *S. aureus*. It is applied for 5–14 days (especially before a surgical intervention) to reduce the risk of postoperative wound infection and prevent the spread of bacteria to medical staff’s hands [182]. Screening of *S. aureus* is mandatory in patients undergoing cardiac or orthopaedic surgery, especially for MRSA. Moreover, all patients prior to surgical procedures must have a full-body shower (with/without disinfectants such as chlorhexidine) to reduce the number of surgical site infections and complications caused by this highly pathogenic and resistant microorganism [183]. The mechanism by which mupirocin exerts its antibacterial activity consists of the inhibition of isoleucyl-tRNA (ended by ileS gene on the chromosome) and, thus, the blocking of protein synthesis within the bacteria. Two types of mupirocin resistance have been described: low-level (MupRL, MIC: 8–256 mg/L) and high-level (MupHR, MIC: 512 mg/L). Usually, the MIC of mupirocin on sensible strains is around 4 mg/L. If MupRL is caused by ileS gene mutation, MupRH is induced by a plasmid encoded in the ileS2 gene (responsible for encoding a different isoleucyl-tRNA synthase) which will trigger a low affinity for mupirocin [182]. In the study performed, carvacrol highlighted the best inhibitory action on the tested MRSA strains and 1,8-cineole induced a synergistic action against MupRL MRSA with penicillin G. The authors suggested that high precision technology that trigger crucial checkpoints for staphylococcal resistance is needed [184] (Appendix B, Table A6).

The group of Manzuoerh et al. investigated the in vivo effect of topical dill (*Anethum graveolens*) EO versus mupirocin on a MRSA-induced infection in a BALB/c mouse model. They used two different concentrations of dill EO and determined that the main active components were α-phellandrene (47.3%), *p*-cymene (18.5%) and carvone (14.1%). The topical administration of dill EO decreased the inflammation and stimulated re-epithelialization, angiogenesis and collagen and fibroblast sedimentation. The topical effects were the result of an increased expression of p53 and caspases-3, in the case of the anti-inflammatory activity, and Blc-2, VEGF and FGF-2 expression in the case of the proliferative activity. Together with the stimulation of collagen synthesis through increases of ERα expression level, all these effects led to improvements in wound healing and reduction of the infection [185]. Mahboubi M et al. performed a similar study on EOs obtained from the aerial parts of *Oliveria decumbens* and *Pelargonium graveolens*. The main active substances in *Oliveria decumbens* EO were thymol (50.1%), *γ* -terpinene (20.7%) and p-cymene (17.6%), while beta-citronellol (39.3%) and geraniol (23.6%) were present in *Pelargonium graveolens* EO. Both the herbal cream containing the two EOs and the mupirocin formulation diminished log CFU (colony-forming units). Although further clinical and toxicological studies are required, topical formulations of EOs could be used in the treatment of wound infections, given their antimicrobial activity and healing effects [186] (Appendix B, Table A6).

In 2020, Chen J and collaborators presented the metabolomics analysis of antibacterial activity of camphor leaves EO (*Cinnamomum camphora*) [187]. It is worth mentioning that metabolomics analysis contributes to a multifactorial description of a drug mechanism of action and identifies eventual dynamic changes in metabolites as a response to drug treatment [188]. Moreover, key marker identification through pathway analysis can be used in order to identify metabolites’ changes in the course of antimicrobial activity. The camphor EO was rich in linalool (26.6%) and eucalyptol (16.8%), as well as α-terpineol (8.7%), isoborneol (8.1%), *β*-phellandrene (5.1%) and camphor (5.0%). The metabolomics analysis revealed 74 different metabolites (29 upregulated and 45 downregulated). The EO stimulated the activity of isocitrate dehydrogenase (47.35%) and decreased the activity of malate dehydrogenase (72.63%), succinate dehydrogenase (31.52%) and α-ketoglutarate dehydrogenase (63.29%). The authors concluded that the antimicrobial activity was the result of an imbalance in the amino acid metabolism, a rise in the apoptosis rate and a disruption in cell wall and membrane with the efflux of DNA, RNA and proteins from the MRSA cell [187] (Appendix B, Table A6).

There is a scarcity of studies describing the antimicrobial properties of EOs against VRSA and VISA strains. Vasconcelos SECB et al. investigated the antibacterial activity of Mexican mint (*Plectranthus amboinicus*) EO on VRSA and oxacillin-resistant *S. aureus* and determined that the strains were more sensitive when both carvacrol and EO were used [189] (Appendix B, Table A6).

Further data from these and other studies are provided in Appendix B, Table A6.

## 4. Conclusions

This systematic review presents the most recent studies of EOs’ activity against pathogens on WHO’s priority 2 list. While the emergence of AMR is a natural evolutionary process in bacteria, the widespread use and misuse of ABs has had an amplifying effect on this process.

Due to their selectivity on pathogenic bacteria and relatively low toxicity, EOs have been proposed as good alternatives and valuable adjuvants in a variety of infections. However, testing and evaluating the antimicrobial activity of EOs is difficult because of their volatility, water insolubility and complexity. Moreover, long incubation times during the testing period may result in the evaporation or decomposition of some of the active components. EOs’ phytocompound composition can vary and is influenced by plant subspecies, geographical location, growing conditions, growth phase, extraction method, and exposure to light, temperature and humidity.

There is a need for a consensus on the methodology used to assess the antimicrobial activity of EOs. In our paper, we refrained from a strict assessment of the results in the reviewed studies, given the heterogeneity of research design and techniques. Further research incorporating standardization methods to form a universal consensus are needed to allow a critical examination of EOs’ antimicrobial action. As a general consideration, some authors interpret the findings as follows: MIC: <100 µg/mL—highly active; MIC = 100–500 µg/mL—active; MIC = 500–1000 µg/mL—moderately active; MIC = 1–2 mg/mL—low activity; and MIC: >2 mg/mL—inactive [141,149].

Interestingly, suggestions that might enable the use of EOs in therapy have been mentioned as limitations in some of studies cited above. Thus, in order to provide cohesive results and to facilitate the use of EOs in clinical practice, the following should be considered: (1) assurance that the techniques used to analyse EOs’ composition are compliant with pharmacopeial requirements; (2) investigation of at least 10 different strains of the same microbial species in the same study, as they vary greatly from one another; (3) inclusion of anti-biofilm activity; (4) implementation of cytotoxicity assays for each study as there are limited data regarding EO dosage and safety, especially in humans; (5) increased standardization in methodology; and (6) intensive monitoring over time. Until further research elicits sufficient data for the large-scale use of EO phytocompounds in the treatment of infection, the general population might incorporate EOs into their daily diet for prophylaxis. For instance, not all cultures take the best benefits from using spices, even though they have good, non-specific and multidirectional antimicrobial activity.

In conclusion, while there is growing evidence for the introduction of EOs into clinical practice, especially those which observed no toxic effects, they are currently underused in practice. Based on their efficacy from the combined action of antimicrobial compounds and their synergistic activity with conventional ABs or food preservatives, EOs have the potential to lessen the burden of AMR. The development of standardized techniques for analysing the in vivo antimicrobial activity of EOs is a critical first step.

## Figures and Tables

**Table 1:** Top 5 countries in terms of AMR to HPPs, according to One Health Trust’s Resistance Map [31].

Bacteria	AB Resistance	Year	Resistant Strains Proportion (%)	Country
*E. faecium*	Vancomycin	2017	69 (62–75)	Argentina
		2016	68 (65–71)	USA
		2016	66 (60–71)	Taiwan
		2019	60 (52–67)	Serbia
		2012	51 (42–60)	Venezuela
*S. aureus*	Methicillin	2019	88 (77–95)	Egypt
		2017	73 (69–77)	Vietnam
		2019	68 (66–69)	India
		2018	66 (60–72)	Nigeria
		2019	65 (59–70)	Pakistan

## Data Availability

All the extracted data is presented within the manuscript.

## Abbreviations

ABs	Antibiotics
AMR	Antimicrobial resistance
ARG	Antibiotic resistant genes
ATP	Adenosine triphosphate
CRHP	Clarithromycin-resistant *H. pylori*
CRNG	Cephalosporin-resistant *N. gonorrhoeae*
EOs	Essential oils
ESBL	Extended spectrum beta-lactamase
ESKAPE	*E. faecium*, *S. aureus*, *K. pneumoniae*, *A. baumanni*, *P. aeruginosa* and *E. cloacae*
EU	European Union
FIC	Fractional inhibitory concentration
FRNG	Fluoroquinolone-resistant *N. gonorrhoeae*
FRS	Fluoroquinolone-resistant *Salmonellae* spp.
GLASS	Global Antimicrobial Resistance Surveillance System
HPP	High priority pathogen
IC50	Half-maximal inhibitory concentration
iNTS	Invasive nontyphoidal salmonellosis
KPC	*K. Pneumoniae* carbapenemase
MBC	Minimum bactericidal concentration
MBEC50%	Minimum biofilm inhibitory concentration
MDR	Multidrug resistant
MIC	Minimum inhibitory concentration
MRSA	Methicillin-resistant *Staphylococcus aureus*
MSSA	Methicillin-sensitive *Staphylococcus aureus*
niNTS	Non-invasive non-typhoidal salmonellosis
NTS	Nontyphoidal salmonellosis
PDR	Pandrug-resistant
PMQR	Plasmid-mediated quinolone resistance
PPI	Proton-pump inhibitor
RNA	Ribonucleic acid
QRDR	Quinolone resistance-determining region
VISA	Vancomycin-intermediate *Staphylococcus aureus*
VREF	Vancomycin-resistant *Enterococcus faecium*
VSEF	Vancomycin-sensitive *Enterococcus faecium*
VRSA	Vancomycin-resistant *S. aureus*
WHO	World Health Organization
XDR	Extensively drug-resistant
ZOI	Zone of inhibition

## Appendix A

**Table A1:** Top 5 European countries in terms of AMR to HPPs, according to the Surveillance Atlas of Infectious Diseases [32].

Bacteria	AB Resistance (Year)	Resistant Strains Proportion (%)	Country
*E. faecium*	Vancomycin (2020)	56.6	Lithuania
		44.2	Cyprus
		41.8	Greece
		40.0	Slovakia
		39.3	Romania
*S. aureus*	Methicillin (2020)	49.1	Cyprus
		47.3	Romania
		40.2	Greece
		33.5	Italy
		29.7	Portugal
*N. gonorrhoeae*	Ciprofloxacin (2019)	100.0	Cyprus
		100.0	Croatia
		80.0	Italy
		78.5	Hungary
		75.0	Estonia
	Cefixime (2019)	50.0	Cyprus
		11.1	Croatia
		3.0	Italy
		2.5	Belgium
		1.8	Portugal
	Ceftriaxone (2019)	0.9	Portugal
		0.6	Belgium
		0.2	Norway
		0.0	Austria
		0.0	Germany

## Appendix B

**Table A2:** Studies assessing the antimicrobial activity of essential oils against vancomycin-resistant *Enterococcus faecium*.

Study Team and Year	Bacterial Strain	Essential Oil(s)	Method(s)	Results	Reference
Saki M et al., 2020	VREF, MRSA	*Cinnamomum zeylanicum*	Agar disc diffusion Broth microdilution	*C. zeylanicum* EO (bark; containing: eugenol, trans-cinnamaldehyde, coumarin, benzaldehyde, diacetone alcohol, benzylcarboxaldehyde, styrene and phenol) showed potent antibacterial effects on the bacterial isolates. The most sensitive isolate was MRSA, followed by VREF (MIC = 0.15–1.25 μL/mL for *S. aureus*; MIC = 0.15–2.5 μL/mL for *E. faecium*).	[63]
Iseppi R et al., 2021	Vancomycin-resistant *Enterococcus* spp., MRSA	*Citrus aurantium* subsp. amara Engler, *Citrus × limon* L. Osbeck, *Eucalyptus globulus* Labill., *Melaleuca alternifolia* (Maiden and Betche) and *Cupressus sempervirens* L. (Mill.)	Agar disc diffusion Broth microdilution	All EOs displayed antibacterial effect against all strains to different extents, but *M. alternifolia* EO was the most effective and *C. aurantium* showed the lowest activity. EO–EO and EO–AB associations showed a synergistic outcome in most tests and were effective against biofilm formation.	[64]
Sakkas H et al., 2018	Vancomycin-resistant *Enterococcus* spp., *MRSA*	*Ocimum basilicum* L. (estragole), *Matricaria chamomilla* L. (bisabolol and trans-b-farnesene), *Thymus capitatus* L. (carvacrol and thymol), *Melaleuca alternifolia* (terpinen-4-oland p-cymene) and *Thymus vulgaris* L. (thymol, linalool and p-cymene)	Broth microdilution	*T. capitatus* EO yielded the best antimicrobial results followed by *T. vulgaris*, *M. alternifolia* and *O. basilicum*, while *M. chamomilla* EO exhibited weak antibacterial properties (MIC for *S. aureus* = 0.06–0.5% (*v*/*v*) for *T. capitatus*; 0.06–1% (*v*/*v*) for *T. vulgaris*; 0.12–1% (*v*/*v*) for *M. alternifolia*; 0.25–4% (*v*/*v*) for *O. basilicum;* and 2->4% (*v*/*v*) for *M. chamomilla*; MIC for *Enterococcus* spp. = 0.25–1% (*v*/*v*) for *T. capitatus*; 0.5–2% (*v*/*v*) for *T. vulgaris*; 1–4% (*v*/*v*) for *M. alternifolia*; >4% (*v*/*v*) for *O. basilicum;* and >4% (*v*/*v*) for *M. chamomilla*).	[67]
Owen L et al., 2019	Vancomycin-sensitive *E. faecium* (VSEF), VREF, MSSA, MRSA	*Origanum compactum* Benth (carvacrol), *Aniba roseadora* Ducke (linalool) and *Cuminum cymimum* L. (cuminaldehyde)	Kirby–Bauer disc diffusion Thin layer chromatography-direct bioautography	*O. compactum* EO exhibited strong antimicrobial activity against *S. aureus* and *E. faecium* (MIC = 0.29–1.15 mg/mL). *C. cymimum* EO was strongly antimicrobial against MSSA and MRSA (MIC = 0.58–2.33 mg/mL), but had weaker activity on *E. faecium* (MIC = 18.60–37.20 mg/mL). *A. roseadora* EO was relatively inactive against *S. aureus* and *E. faecium* (MIC = 8.80–35.20 mg/mL).	[66]
Owen L et al., 2020	VSSA, VREF	*Origanum compactum* Benth (carvacrol), *Aniba roseadora* Ducke (linalool) and *Cuminum cymimum* L. (cuminaldehyde)	Checkerboard method Time kill assay Transcriptomic analysis Gradient plate method Sodium chloride tolerance Galleria mellonella treatment assays β-galactosidase leakage	The EO–vancomycin combination is able to kill clinical isolates of VRE (2.33–5.25 log10 CFU/mL reduction). However, in vivo *G. mellonella* larvae assay showed no antimicrobial activity of the EO–vancomycin combination.	[65]
Di Vito M et al., 2021	Methicillin-sensitive *E. faecium*, MRSA	*Lavandula angustifolia*, *Lavandula intermedia*, *Origanum hirtum*, *Satureja montana*, *Monarda didyma*, *Monarda fistulosa*	Broth microdilution	*S. montana* and *O. hirtum* EOs exhibit the highest activity (IR90 = 0.25–1% *v*/*v*). *O. hirtum* and *M. didyma* hydrolates were more active than the other three (IR90 = 50% *v*/*v*). Hydrolates need to be 25–200 times more concentrated than EOs to reach the same antimicrobial activity.	[68]

**Table A3:** Studies assessing the antimicrobial activity of essential oils against clarithromycin-resistant *Helicobacter pylori*.

Study Team and Year	Bacterial Strain	Essential Oil(s)	Method(s)	Results	Reference
Elkousy R et al., 2022	*H. pylori* ATCC 43504	*Origanum majorana* L. (marjoram) *Citrus reticulata* L. (mandarin)	Micro-well dilution	*O. majorana* is more effective against *H. pylori* (MIC = 11.4 mg/mL), while *C. reticulata* possess a slightly lower antibacterial activity (MIC = 31.25 mg/mL). The combination of the two EOs exhibited a synergistic inhibitory effect against *H. pylori* (MIC = 1.95 mg/mL).	[83]
Al-Sayed E et al., 2021	*H. pylori* RCMB 031124, ATCC 43504	*Piper nigrum* L. (black and white pepper) *Piper longum* L. (long pepper) *Piper cubeba* L.F. (tailed pepper)	Micro-well dilution	*P. longum* EO had the highest anti-*H. pylori* activity (MIC = 1.95 μg/mL). White pepper EO was next in terms of antimicrobial effect (MIC = 3.90 μg/mL), followed by *P. cubeba* and *P. nigrum* EO (MIC = 7.81 μg/mL).	[84]
Meriem M et al., 2016	*H. pylori* isolated from gastric biopsy	*Pistacia lentiscus* var. Chia (mastic tree)	Disc diffusion	*P. lentiscus* showed a strong antimicrobial activity, confirmed by the MIC (1/5000, *v*/*v*) and the inhibition diameters (32 mm, 26.66 mm, 19.67 and 12.33 for the pure and diluted oil to 1/2, 1/4 and 1/8). The effect on *H. pylori* can be attributed to its content in monoterpenes (α-pinene, limonene and *β*-myrcene).	[86]
Knezevic P et al., 2018	Metronidazole-resistant *H. pylori*, *H. pylori* ATCC 26695 (ATCC 700392)	*Juniperus communis* L., *Hyssopus officinalis* L., *Salvia officinalis* L., *Melissa officinalis* L., *Lavandula angustifolia* Mill., *Ocimum basilicum* L. *Thymus serpyllum* L.	Broth microdilution	*J. communis*, *H. officinalis* and *O. basilicum* EOs did not show any antibacterial activity, with the highest applied concentrations. *S. officinalis* EO inhibits growth of *H. pylori* ATCC 26695 (MIC = 4 µL/mL), while *L. angustifolia* and *M. officinalis* EOs have anti-*H. pylori* activity depending on oil composition and strain properties. The most active EO was *T. serpyllum* (MIC = 2.0–4.0 µL/mL).	[87]
Ali S et al., 2022	*H. pylori* isolated from gastric biopsy	*Syzygium aromaticum* L. (clove) *Thymus vulgaris* L. (thyme) *Rosmarinus officinalis* L. (rosemary) *Matricaria recutita* L. (chamomile) *Cinnamomum zeylanicum* L. (cinnamon)	Disc diffusion Agar well diffusion Cytotoxicity test	*C. zeylanicum* EO has the strongest anti-*H. pylori* activity, with a mean inhibition zone of 23.4 mm, higher than the levofloxacin positive control. Mean inhibition zones for *S. aromaticum*, *T. vulgaris* and *R. officinalis* EOs were 19.8 mm, 15.5 mm and 9.8 mm, respectively.	[88]
Korona-Glowniak I et al., 2020	*H. pylori* ATCC 43504 *H. pylori* isolated from clinical settings (both sensitive and resistant)	*Pinus sylvestris* L. (pine needle), *Citrus lemon* L. (lemon), *Abies alba* Mill (silver fir), *Thymus vulgaris* L. (thyme), *Cymbopogon schoenanthus* (L.) Spreng (lemongrass), *Juniperus virginiana* L. (cedarwood) *Melissa officinalis* L. (lemon balm), *Melaleuca alternifolia* Maiden et Betche (tea tree), *Origanum vulgare* L. (oregano)	Urease inhibition activity Antioxidant activity analysis Broth microdilution	The most active EOs were *T. vulgaris*, *C. schoenanthus*, *J. virginiana* and *M. officinalis* (MIC = 15.6 mg/L), followed by *O. vulgare* (MIC = 31.3 mg/L), *M. alternifolia* (MIC = 62.5 mg/L) and *P. sylvestris*, *A. alba*, and *C. lemon* (MIC = 125 mg/L) EOs. Regarding urease inhibition activity, the most efficient EO was *J. virginiana* EO (IC50 = 5.3 mg/L), inhibiting urease at sub-MIC concentrations.	[89]
Gad H. et al., 2021	*H. pylori* RCMB 031124, ATCC 43504	*Pinus canariensis* C. Sm. ex D.C., *Pinus halepensis* Miller, *Pinus pinea* L. *Pinus roxburghii* Sarg.	Well dilution	*P. pinea* EO exhibited the highest antimicrobial activity (MIC = 3.9 μg/mL), comparable to that of clarithromycin. *P. halepensis*, *P. roxburghii* and *P. canariensis* EOs showed a milder anti-H. pylori activity (MIC = 15.6 μg/mL).	[85]
Neves NCV C et al., 2022	Clarithromycin-sensitive *H. pylori*, CRHP—type cultures and clinical isolate strains	*Campomanesia lineatifolia* R. and P.	Broth microdilution	*C. lineatifolia* EO inhibits the growth of all *H. pylori* strains at the lowest concentration tested (MIC = 6 µL/mL).	[90]
Bhattamisra S et al., 2019	*H. pylori* ATCC 43504	Geraniol (active component found in the lemongrass, rose, palmarosa, ginger, orange, lavender, citronella and nutmeg EOs)	Measurement of gastric juice volume, pH and total acidity Determination of myeloperoxidase Determination of total glutathione Rapid urease test Histopathological evaluation	Geraniol presents antiulcer effect and anti-*H. pylori* action, associated with decreased myeloperoxidase activity, gastric secretion and bacterial load, as well as increased glutathione levels and gastric pH.	[91]
Memariani Z et al., 2017	Clinical *H. pylori* strains	*Pistacia atlantica* Desf.	Disc diffusion assay Micro-well dilution assay Microscopic evaluation of gastric ulcer Measurement of ulcer index and calculation of protection rate Acute toxicity	*P. atlantica* EO (rich in α-pinene) was safe up to 2000 mg/kg and no clinical sign of toxicity was observed. All *H. pylori* strains were sensitive to this EO (inhibition zone = 26–35 mm, MIC = 275–1100 µg/mL). The highest dose of EO (100 mg/kg) protected significantly better against peptic ulcer than that of the ranitidine treated group.	[92]
Jung D et al., 2020	*H. pylori* KCTC12083	*β*-caryophyllene (active compound found in cloves, basil, cinnamon, and copaiba)	Histopathological assessment In vitro quantification of colony forming units	*β*-caryophyllene dose-dependently diminished *H. pylori* levels and inflammation in gastric mucosa. In vitro test determined that a concentration of 1 M *β*-caryophyllene was able to eradicate 99.9% of *H. pylori* strains but other doses did not have the same antimicrobial efficacy.	[93]

**Table A4:** Studies assessing the antimicrobial activity of essential oils against fluoroquinolone-resistant *Campylobacter* spp.

Study Team and Year	Bacterial Strain	Essential Oil(s)	Method(s)	Results	Reference
Gahamanyi N et al., 2020	*C. jejuni* strains ATCC 33560TM and MT947450 *C. coli* strains ATCC 33559TM and MT947451	*Cinnamomum cassia* (L.) J. Presl (cinnamon) extract and EO, *Salvia plebeia* R. Br (common sage) extract, *Mentha canadensis* L. (wild mint) extract, *Scutellaria baicalensis* Georgi (skullcap) extract, *Meehania urticifolia* (Miq.) Makino (nettle-leaf mint) extract, *Syzygium aromaticum* L. (clove) EO, (E)-Cinnamaldehyde, eugenol, baicalein, kuraridin and emodin	Broth microdilution	The highest anti-*Campylobacter* activity was recorded for *C. cassia* EO and phytochemical (E)-cinnamaldehyde (MIC = 25–50 µg/mL). Clove oil and its major compound eugenol showed as well activity against *Campylobacter* spp. (MIC = 50–100 µg/mL). Other active compounds displayed good antimicrobial effects: baicalein (MIC = 32–64 µg/mL) and kuraridin (MIC = 48 µg/mL), while emodin had the lowest activity (MIC = 50 µg/mL for *C. jejuni* and 200 µg/mL for *C. coli*).	[107]
Imunović K et al., 2019	*C. jejuni*	(-)-*α*-pinene	Broth microdilution Quorum-sensing inhibition in vitro Broiler chicken colonization with *C. jejuni* broiler	By itself, (-)-*α*-pinene showed low antimicrobial activity (MIC50 = 2000 mg/L); however, when in combinations, (-)-*α*-pinene was able to reduce ciprofloxacin’s and erythromycin’s MICs. When treating cultures with three subinhibitory concentrations of (-)-*α*-pinene, a reduction in quorum-sensing signalling molecules is observed. However, (-)-α-pinene did not manage to impede fluoroquinolone resistance development when added to enrofloxacin in broiler chickens.	[114]
Lin L et al., 2018	*C. jejuni*	*Thymus vulgaris* L. (thyme)	Plate count Measurement of inhibition zone Sodium dodecyl sulphate-polyacrylamide gel electrophoresis (SDS-PAGE) Transmission electron microscopy (TEM)	The population of *C. jejuni* was lower in the group treated with thyme EO *β*-cyclodextrin *ε*-polylysine nanoparticles (TCPNs) than in control group, both in chicken soup (difference = 2.74 log CFU/mL, inhibition zone = 25.10 mm) and commercially available chicken meat (difference = 1.38 log CFU/g). Due to the encapsulation and presence of ε-polylysine, the prepared TCPNs exhibited better and prolonged anti-*C. jejuni* activity than thyme EO/*β-*cyclodextin inclusion complex and free thyme EO. Damage in cell membrane and protein leakage in *C. jejuni* treated with TCPNs were emphasized by TEM and SDS-PAGE analysis.	[112]
Duarte A et al., 2016	*C. jejuni* ATCC 33560 and 225421, *C. coli* ATCC 33559 and 873	*Coriandrum sativum* L. (coriander), linalool	Broth microdilution Anti-biofilm activity Disc diffusion assay Vapour diffusion assay Broth assay Evaluation of the antioxidant activity Anti-quorum-sensing activity	Both EO and linalool showed anti-*Campylobacter* activity (diameter of inhibition > 85 mm, MIC = 0.5–1 µL/mL), inhibited biofilm formation and produced quorum-sensing inhibition.	[108]
Kovács J et al., 2016	C. jejuni NCTC 11168	*Syzygium aromaticum* L. (clove), eugenol, beta-caryophyllene and alpha-humulene	Broth microdilution Time kill assay	*S. aromaticum* EO (MIC = 200 µg/mL and MBC = 800 µg/mL) and eugenol showed antimicrobial properties and altered *C. jejuni* morphology, whereas beta-caryophyllene and alpha-humulene had no such effects.	[109]
Ahmed J et al., 2016	C. jejuni ATCC 33291 S. aureus ATCC 6538	*Cinnamomum cassia* Presl (cinnamon), *Allium sativum* L. (garlic) and *Syzygium aromaticum* L. (clove) EOs	Agar disc diffusion	Zone of inhibition showed that both *S. aureus* and *C. jejuni* were sensitive to clove and cinnamon EOs, while garlic EO was found to be less effective. Same outcome resulted from survivor counts (CFU/mL).	[110]
El Baaboua A et al., 2022	MDR Campylobacter spp.	*Origanum compactum* Benth (oregano), *Rosmarinus officinalis* L. (rosemary), *Mentha pulegium* L. (mint) and *Lavandula stoechas* L. (lavender)	Agar well diffusion Broth microdilution Biofilm detection	Diameters of inhibition showed that *O. compactum* and *L. stoechas* EOs had high anti-campylobacterial effect (diameter = 15 -> 80 mm and 24 -> 80 mm, respectively). High sensitivity of Campylobacter spp. toward *O. compactum*, *M. pulegium* and *L. stoechas* was also sustained by MIC, with the lowest value recorded of 0.063% (*v*/*v*).	[111]
Lin L et al., 2019	*C. jejuni* CICC 22,936	Chrysanthemum	Transmission electron microscopy (TEM)	The *C. jejuni* population of treated samples decreased from 3.2 log CFU/mL to 0 log CFU/mL at 12 °C for 12 days. However, at 25 °C and 37 °C the population of *C. jejuni* was around 1.2 log CFU/mL to 2.3 log CFU/mL.	[113]

**Table A5:** Studies assessing the antimicrobial activity of essential oils against cephalosporin-resistant, fluoroquinolone-resistant *Neisseria gonorrhoeae*.

Study Team and Year	Bacterial Strain	Essential Oil(s)	Method(s)	Results	Reference
Vică M et al., 2021	Ciprofloxacin-sensitive *N. gonorrhoeae*, Ciprofloxacin-intermediate-resistant *N. gonorrhoeae*, Ciprofloxacin-resistant *N. gonorrhoeae*,	Aqueous extracts of propolis samples	Disc diffusion Broth microdilution	The propolis extracts possessed anti-*N. gonorrhoeae* activity. The mean diameter of the inhibition zones for propolis extracts was 39.75 mm (27–42 mm), in some cases exceeding ciprofloxacin’s zone of inhibition. For most extracts, MIC was 6.25 µg/mL, while some propolis samples exhibited MIC of 12.5 µg/mL or 25.0 µg/mL.	[130]
Umaru I et al., 2020	MDR *S. aureus* MDR *N. gonorrhoea*	*Molineria capitulata* Lour., myrcene	Disc diffusion	At 500 µg/mL, *M. capitulata* EO exhibited ZOI = 23.28 ± 0.13 mm on *S. aureus* and ZOI = 22.3 ± 0.37 mm on *N. gonorrhoeae*. Myrcene showed similar results, with ZOI = 23.53 ± 0.13 mm on *S. aureus* and ZOI = 20.50 ± 0.17 mm on *N. gonorrhoeae*.	[131]
Soliman F et al., 2016	*Neisseria gonorrhoeae* 19424, *Staphylococcus aureus* ATCC 12600	*Psidium guajava* L. (guava leaf), *Psidium cattleianum* Sabine (strawberry guava)	Agar disc diffusion Agar dilution	*S. aureus*: ZOI = 16 ± 0.15 mm (*P. guajava*) and 10 ± 0.28 mm (*P. cattleianum*); MIC = 6.75 µg/mL (*P. guajva*). *N. gonorrhoeae*: ZOI = 12 ± 0.22 mm (P. guajava) and 13 ± 0.29 mm (*P. cattleianum*); MIC = 13.01 µg/mL (*P. cattleianum*).	[132]
Zaman G et al., 2021	*N. gonorrhoeae*, *S. aureus*	*Eclipta alba* L. (kenharaj), *Atriplex hortensis* L. (pahari palang), *Hedyotis scandens* (Roxb.) (bhedeli-lota), *Leucas linifolia* Spreng (doron bon), *Murraya koenigii* L.(narasingha), *Phlogacanthus thyrsiflorus* Nees (tita-phul)	Disc diffusion Broth microdilution	The antimicrobial activity varied as follows: *E. alba* (for *N. gonorrhoeae*: ZOI = 22.60 ± 2.50 mm, MIC = 1.50 ± 0.20 μg/mL, MBC = 3.06 ± 0.40 μg/mL; for *S. aureus*: ZOI = 17.20 ± 1.47 mm, MIC = 0.05 ± 0.02 μg/mL, MBC = 0.11 ± 0.03 μg/mL); *A. hortensis* (for *N. gonorrhoeae*: MIC = 6.0 ± 0.26 μg/mL, MBC = 12.07 ± 0.65 μg/mL; for *S. aureus*: ZOI = 12.90 ± 2.55 mm, MIC = 3.53 ± 0.35 μg/mL, MBC = 8.73 ± 2.62); *H. scandens* (for *N. gonorrhoeae:* ZOI = 12.60 ± 1.43 mm, MIC = 7.50 ± 0.36 μg/mL, MBC = 15.30 ± 0.43 μg/mL; for *S. aureus*: ZOI = 20.50 ± 3.10 mm, MIC = 6.66 ± 0.40 μg/mL, MBC = 13.0 ± 0.65); *L. linifolia* (for *N. gonorrhoeae:* ZOI = 18.20 ± 1.22 mm, MIC = 3.50 ± 0.20 μg/mL, MBC = 7.06 ± 0.40 μg/mL; for *S. aureus*: ZOI = 8.0 ± 0.81 mm, MIC = 4.33 ± 0.25 μg/mL, MBC = 8.73 ± 0.61); *M. koenigii* (for *N. gonorrhoeae*: ZOI = 15.50 ± 1.08 mm, MIC = 5.46 ± 0.20 μg/mL, MBC = 11.07 ± 0.37 μg/mL; for *S. aureus*: ZOI = 7.40 ± 1.17 mm, MIC = 6.10 ± 0.43 μg/mL, MBC = 12.50 ± 0.79); and *P. thyrsiflorus* (for *N. gonorrhoeae:* ZOI = 18.40 ± 0.96 mm, MIC = 5.10 ± 0.17 μg/mL, MBC = 10.20 ± 0.34 μg/mL; for *S. aureus*: ZOI = 18.30 ± 0.82 mm, MIC = 4.70 ± 0.20 μg/mL, MBC = 9.40 ± 0.40)	[133]
Elghwaji W et al., 2017	*N. gonorrhoeae* ATCC 19424, *S. aureus* ATCC 12600	*Ferula tingitana* L.	Agar disc diffusion Evaluation of cytotoxic activity	*N. gonorrhoeae*: ZOI = 12 mm (*F. tingitana* flower-derived EO), ZOI = 13 mm (*F. tingitana* leaves-derived EO); *S. aureus*: ZOI = 9 mm (*F. tingitana* flower-derived EO), ZOI = 11 mm (*F. tingitana* leaves-derived EO). IC50 for *F. tingitana* flower-derived EO = 6.9 µg/mL, 8.6 µg/mL and 4.4 µg/mL; for *F. tingitana* leaves-derived EO = 4.8 µg/mL, 4.2 µg/mL and 10.9 µg/mL, on breast, liver and cervical carcinoma cell lines. The results were comparable to the standard cytotoxic drug doxorubicin.	[134]

**Table A6:** Studies assessing the antimicrobial activity of essential oils against methicillin resistant, vancomycin-intermediate and -resistant *S. aureus* (a non-exhaustive list).

Study Team and Year	Bacterial Strain	Essential Oils	Method(s)	Results	Reference
Ben Abdallah F et al., 2020	MRSA	*Origanum majorana* L. (origanum), *Rosmarinus officinalis* L. (rosemary) and *Thymys zygis* L. (common thyme)	Disc diffusion MIC and MBC methods Crystal violet	Potent antimicrobial effect shown by all tested EOs, *T. zygis* having the most powerful effect (MIC = 0.39 mg/mL–0.78 mg/mL; MBC = 3.125 mg/mL), followed by *O. majorana* (MIC = 0.78 mg/mL–1.56 mg/mL; MBC = 3.125 mg/mL–12.5 mg/mL) and *R. officinalis* (MIC = 0.78 mg/mL–1.56 mg/mL; MBC = 12.5 mg/mL). Regarding the biofilm inhibition and eradication effect, *O. majorana* EO presented the most potent activity (inhibition effect: from 10.29 to 95.91% and eradication effect: from 18.31 to 98.01%).	[190]
Gomez-Sequeda N et al., 2020	MRSA *E coli* O157:H7	*Lippia origanoides* Kunth	Broth microdilution Cytotoxicity assay Scanning electron microscopy	Thymol and carvacrol chemotypes from *Lippia origanoides* exhibited the best antimicrobial action for MRSA (MIC = 1.2 and 0.6 mg/mL) and for *E. coli* O157:H7 (MIC = 0.9 and 0.3 mg/mL), as well as the best antibiofilm inhibition (>70.3%), but the analysis performed on the selectivity index (≤3) highlighted the fact that further studies are required in order to reduce its in vitro toxicity.	[191]
Ekhtelat M. et al., 2020	MRSA *Yersinia enterocolitica*	*Cuminum cyminum* L. (cumin) *Mentha spicata* L. (spearmint) *Mentha longifolia* L. (horse mint) Single or in combination with sodium benzoate	Agar disc diffusion Micro-well dilution assay	*M. longifolia* L. (main component: pulegone) and *C. cyminum* L. (main component: aldehyde) presented the best antibacterial effect against the tested strains. The association with sodium benzoate exhibited a more potent antibacterial effect, compared with the use of sodium benzoate alone as antimicrobial agent, and therefore can reduce the high doses required if used alone for preservation of food or drug products, and thus its toxicity.	[192]
Tang C. et al., 2020	MRSA	*Amomum villosum* Lour	Label-free quantitative proteomics Sodium dodecyl sulphate-polyacrylamide gel electrophoresis analysis Scanning electron microscopy	The antibacterial effect expressed by the tested EO demonstrated its mechanism of action: cell membrane lesion which affects its integrity, intracellular leakage of substances, protein inhibition and biofilm synthesis inhibition.	[193]
Rubini D. et al., 2018	MRSA	*Pogostemon heyneanus* Benth. *Cinnamomum tamala* Nees and Eberm (Indian bay leaf)	Confocal laser scanning microscopy Scanning electron microscopy	Both EOs affected the MRSA preformed biofilms and were successful in reducing virulence factors such as staphyloxanthin and hemolysin, with (*E*)-nerolidol having a higher affinity for dehydroxysqualene synthase (responsible for the synthesis of staphyloxanthin).	[194]
Utegenova GA et al., 2019	MRSA	*Ferula ovina* (Boiss.) Boiss, *Ferula iliensis* Krasn. ex. Korovin, *Ferula akitschkensis* B. Fedtsch. Ex Koso-Pol.	Pulse-field gel electrophoresis Colony count technique Broth microdilution	*F. ovina* EOs (from roots and stems at fruiting stages) expressed the most powerful antibacterial activity dependent of the concentration, with IC50 = 19.1, 20.9 and 22.9 mcg/mL. The main components with antibacterial activity being eremophilene and *trans*-verbenol (single or associated), although they were not the major constituents of the EOs.	[195]
Oo T et al., 2021	MRSA	*Myristica fragrans* Houtt. (nutmeg) crude extract and EO	Disc diffusion PCR Checkerboard titration assay	The association of nutmeg preparations with ciprofloxacin led to synergistic action on efflux pump system (chromosomal *nor*A and *mep*A) in MRSA, *mep*A being incriminated for the efflux pump inhibition of EO.	[169]
Sreepian A et al., 2022	MRSA MSSA	*Citrus reticulata* Blanco (mandarin orange) *Citrus* × *aurantiifolia* (Christm.) Swingle (key lime) alone and in combination with gentamicin	Agar disc diffusion Resazurin-based microdilution Checkerboard titration assay	Both EOs manifested inhibitory effects against the tested strains. The most potent antimicrobial activity was observed for *C. reticulata* EO and limonene (the major compound of both EOs). Synergistic effect with gentamicin was observed for *C. reticulata* EO (FIC index = 0.012–0.258) and limonene (FIC index = 0.012–0.375) on both tested strains.	[196]
Dalli M et al., 2021	MRSA *E. coli* *P. aeruginosa* *A. baumannii*	*Nigella sativa* L. (black caraway/cumin)	Agar diffusion method Microdilution method	The tested EOs, although from 4 different countries (India, Saudi Arabia, Morocco, Syria), had similar compositions and were slightly more potent against MRSA compared with the Gram-negative bacteria tested (MIC/MBC = 3–10 µg/mL).	[197]
Alharbi NS et al., 2016	MRSA and oxacillin-resistant *S. aureus*	*Piper cubeba* L. f. (tailed pepper)	Atomic force microscopy Transmission electron microscopy Microdilution assay	Severe damage of *S. aureus* ATCC 43300 cells was observed at microscopic levels with 50 mcg/mL EO when compared to 25 mcg/mL, although the latter produced important modification within the cell wall, at nanoscopic levels. Therefore, both EOs exhibited antibacterial effects on the cell wall, as well as on the plasma membrane.	[170]
Tang C et al., 2021	MRSA	*Amomum villosum* Lour	Metabolomics analysis	The tested EO induced an antibacterial effect by blocking the amino acid metabolism and tricarboxylic acid cycle (the activity of key enzymes was inhibited) of MRSA. Moreover, it inhibited the synthesis of reactive oxygen species and adenosine triphosphate, leading to bacterial cell death.	[198]
Piasecki B et al., 2021	MRSA	*Cymbopogon* spp.	Microdilution Direct bioautography *Danio rerio* “Zebrafish” model assay Broth microdilution assay	*C. flexuosus* (lemongrass EO) expressed the highest antibacterial activity from all the 19 tested EOs of *Cymbopogon* spp. (MIC/MBC = 0.5 mg/mL). Citronellol showed powerful antibacterial activity (MIC/MBC = 0.25 mg/mL). Antibiofilm effects were also observed for all tested EOs (MBIC = 1 mg/mL–4 mg/mL). *C. martini* var. *motia* expressed the most toxic potential (about 20 times more toxic than *C. winterianus*) on zebrafish model assay.	[171]
Iseppi R et al., 2021	MRSA vancomycin-resistant enterococci ESBL-producing *E. coli*	*Citrus* × *aurantium* L. (bitter orange) *Citrus* x *limon* L. (lemon) *Eucalyptus globulus* Labill. (blue gum) *Melaleuca alternifolia* (Maiden and Betche) Cheel (tea tree) *Cupressus sempervirens* L. (Italian cypress)	Agar disc diffusion MIC assay Checkerboard method	Tea tree oil (*M. alternifolia*) was the most effective EO, although all tested EOs presented antibacterial activity. Synergistic action was observed when EOs were associated with other EOs or with classical ABs. Good antibiofilm activity was observed when the EOs were used in monotherapy or in combination	[64]
Merghni A et al., 2018	MRSA	*Eucalyptus globulus* Labill. (blue gum) and 1,8-cineole	Test tube method Crystal violet staining assay Disc diffusion test MIC/MBC methods	*E. globulus*, as well as its main component: 1,8 cineole, presented high antibiofilm activity, with *E. globulus* EO having a better anti-quorum-sensing potential (even at low concentrations) (MBC < 50 mg/mL) than 1,8-cineole alone (MBC > 50 mg/mL).	[172]
Al-Maharik N et al., 2021	MRSA *S. aureus* ATCC 25923 *E. faecium* ATCC 700221 *K. pneumoniae* ATCC 13883 Proteus vulgaris ATCC 700221 E. coli ATCC 25922 *P. aeruginosa* ATCC 27853 *C. albicans* ATCC 90028	*Satureja nabateorum* (Danin and Hedge) Bräuchler	Broth microdilution assay Cell culture cytotoxicity assay	Both the fresh and the air-dried EOs of *S. nabateorum* presented good and similar antimicrobial and fungicidal activity. EO obtained from the air-dried sample manifested a higher antimicrobial activity against MRSA (MIC = 6.25 µg/mL) than ciprofloxacin (MIC = 12.5 µg/mL). Both EOs showed cytotoxic activity against HeLa and HepG2 cancer cells and were proposed as potential alternatives to bactericides and fungicides of chemical origin, as well as natural preservatives and conservation substances.	[199]
Khamis AS et al., 2021	MRSA ATCC 33591 *E. coli* NCTC 10418 *P. aeruginosa* NCTC 10662 *Bacillus subtilis* ATCC 6059 *Micrococcus luteus* ATCC 9341 *S. aureus* NCTC 6571	*Juniperus chinensis* L. (Chinese juniper) *Juniperus seravschanica* Kom. (Pashtun juniper) versus their methanolic crude extracts	Disc diffusion	Although all EOs tested showed antimicrobial activity, increased activity was observed against *M. luteus* and *B. subtilis*. Only the crude methanolic extracts manifested an increased antibacterial activity against MRSA and *S. aureus*. A higher antimicrobial activity was observed for methanolic crude extract of *J. seravschanica* (from Oman) against MRSA.	[173]
Predoi D et al., 2018	MRSA *S. aureus* ATCC 0364 *E. coli* ATCC 25922	Hydroxyapatite coated with basil (Hap-B) Hydroxyapatite coated with lavender (HAp-L) EOs	Fourier transform infrared spectroscopy Broth microdilution	HAp-L was found to exhibit the highest inhibitory growth activity against the tested strains, with MIC = 0.039 mg/mL for MRSA and *E. coli* ATCC 25922, and 0.02 mg/mL for *S. aureus*.	[174]
Mouwakeh A et al., 2019	MRSA MSSA	*Nigella sativa* L. (black caraway) Thymoquinone Carvacrol p-cymene	Broth microdilution Ethidium bromide accumulation assay Real-time reverse transcriptase quantitative PCR Crystal violet assay	The tested strains were susceptible to *N. sativa* EO, carvacrol and thymoquinone (but not p-cymene), and each of the tested compound affected the membrane integrity of MRSA (including p-cymene). P-cymene was found to down-regulate the expression of EP gene *mepA* in MRSA, therefore decreasing its virulence.	[177]
Donadu MG et al., 2020	MRSA MSSA *S. epidermidis* *E. faecalis* *Candida tropicalis* *Candida albicans* *Candida glabrata* *Candida epidermidis* *C. parapsilosis* *E. coli* *P. aeruginosa* *K. pneumoniae* *T. vaginalis* strain G3 Enterovirus—A71 strain	*Hornstedtia bella* Skornick	Broth microdilution Cell and cytotoxic assay	MIC/MLC = 1–4% *v*/*v* for MRSA, MSSA, S. *epidermidis* (β-Pinene, E-β-caryophyllene and α-humulene); MIC/MLC = 2–16% *v*/*v* for *C. tropicalis* and *C. parapsilosis*; MIC/MLC = 4–16% *v*/*v* for *E. faecalis*; and MIC/MLC = 8–16% *v*/*v* for the remaining tested strains. Low cytotoxicity against Vero 76 and MRC-5 for leaf oil, without any toxic effect for rhizomes and whole-plant oils on the cells, as well as no action against enterovirus (EV-A71).	[200]
Bay M et al., 2019	MRSA *E. coli* *P. aeruginosa* *S. mutans* *S. pyogenes* *Trypanosoma cruzi*	*Bocageopsis multiflora* R.E.Fr., *Duguetia quitarensis* Benth., *Fusaea longifolia* (Aubl.) Saff., *Guatteria punctata* (Aubl.) RAHoward	Broth microdilution Trypanocidal activity assays Cytotoxicity assays	Potent antimicrobial activity manifested by the tested EOs against *S. mutans* (MIC = 4.68–37.5 µg/mL. *G. punctata* EO was the most effective against *T. cruzi*, therefore having a good trypanocidal activity, 34 times more active than the reference drug benznidazole (IC50 = 0.029 µg/mL, SI = 32)	[201]
Aelenei P et al., 2019	MRSA MSSA *S. epidermidis* *P. aeruginosa* *E. coli*	*Coriandrum sativum* L. (coriander) and linalool, both associated with ABs	Broth microdilution Checkerboard assay	Synergistic interactions were observed between coriander EO and its major component, linalool, with ABs such as amoxicillin, oxacillin, gentamicin, tetracycline and ciprofloxacin, their MICs being drastically reduced. FICI ≤ 0.5 was obtained for coriander EO + gentamicin or amoxicillin against MRSA.	[202]
Leal ALAB et al., 2021	*S. aureus* SA1199B (overexpressing *nor*A gene) *S. aureus* K2068 (overexpressing *mep*A gene) *S. aureus* K4100 (overexpressing *qac*C gene) *C. albicans* ATCC 10231 *E. coli* ATCC 25922 *S. aureus* ATCC 25923	*Piper caldense* C.DC. alone or in combination with norfloxacin	Microdilution	Antimicrobial effect against *S. aureus* strains was observed only in association with norfloxacin, effect observed at subinhibitory concentrations. In contrast, the EO was active against *C. albicans*, inhibiting an important mechanism of virulence of the fungi, the hypae formation, therefore having a good antifungal activity. The EO was able to act as efflux pump inhibitor on *nor*A, *mep*A and *qac*C.	[203]
Predoi D. et al., 2018	MRSA 1144 *S. aureus* 1426 ESBL *E. coli* 4493 *E. coli* ATCC 25922	*Ocimum basilicum* L. (basil) *Lavandula augustifolia* Mill (lavender) (linalool being the major compound in both EOs)	Broth microdilution Flow cytometric assay	Lavender EO expressed a good antibacterial action (MIC < 0.1% mg/mL for *E. coli* strains and up to 0.78% mg/mL for *S. aureus* strains; MBC < 0.1% mg/mL up to 1.56% mg/mL). The hydroxyapatite solution with lavender EO expressed an increased antibacterial activity (MIC = 0.31 mg/mL; MBC = 0.62 mg/mL for MRSA 1144), making hydroxyapatite a possible vehicle for lavender EO solutions in low concentrations.	[175]
de Jesus GS et al., 2020	*S. aureus* NEWP0023 *E. coli* NEWP 0022 MRSA *mecA* mediated *S. warneri* β-lactamase producer *S. intermedius mecA* mediated methicillin resistance	*Pectis substriata* Rusby alone or in combinations with ABs	Broth microdilution Checkerboard microtiter test	The EO exhibited antibacterial activity depending on the strains with potent antimicrobial activity against *S. warne-ri* and moderate activity against *S. aureus* standard strain and *S. intermedius*. Synergistic actions were observed when associated with ampicillin and kanamycin.	[204]
Cui ZH et al., 2021	MRSA 43300 *E. coli* ATCC 25922 *S. typhimurium* ATCC 14028 *K. pneumoniae* ATCC 700603	29 plant EOs	Modified well diffusion Checkerboard assay Standard time-killing assay	Orange oil + amikacin and petitgrain oil + tetracycline exhibited synergistic actions against *S. aureus*, *S. typhimurium* and *K. pneumoniae*. The same action was observed for petitgrain EO + tetracycline against *E. coli* tested strain.	[205]
de Moura DF et al., 2021	MRSA—clinical isolate MSSA—clinical isolate *P. aeruginosa*—clinical isolate *E. faecalis* ATCC 14 506 *E. coli* ATCC 25 922 *K. pneumoniae* ATCC 29 665 *P. aeruginosa* ATCC 9029 *S. aureus* ATCC 6538 *S. epidermidis* ATCC 12 228 *S. mutans* ATCC 10 499	Nerolidol	Microdilution Crystal violet method	Nerolidol was effective against MRSA (MIC = 2 mg/mL), *P. aeruginosa* and *K. pneumoniae* carbapenemase (MIC = 0.5 mg/mL). It showed dose-dependent antioxidant, antibacterial and antibiofilm activity (the percentage of inhibition being 51–98% at concentrations varying from 0.5 to 4 mg/mL).	[178]
Jaradat N et al., 2021	MRSA *S. aureus* ATCC 25923 *C. albicans* ATCC 90028 *P. aeruginosa* ATCC 9027 *E. coli* ATCC 25922 *K. pneumonia* ATCC 13883 *P. vulgaris* ATCC 8427	*Aloysia citriodora* Palau (lemon verbena)	Broth microdilution assay	Two EOs were obtained from lemon verbena (Umm al-Fahm and Bawa al-Gharbiyye). *α*-Citral (geranial) was determined to be their main component. Potent antimicrobial activity was observed against MRSA and *P. vulgaris* (MIC = 2.5 µg/mL), using ciprofloxacin and ampicillin as reference. Good antifungal activity was noticed against *Candida albicans* (MIC = 0.312–0.625 µg/mL), where fluconazole was used as reference. The cytotoxicity was tested on HeLa tumour cells versus doxorubicin: at a concentration of 500 µg/mL, the viability was 98.13 and 96.09% for the two EOs. The EO from Baqa al-Gharbiyye highlighted a more potent cytotoxic activity (IC50 = 84.5 ± 0.24 µg/mL) vs. doxorubicin (IC50 = 22.01 ± 1.4 µg/mL), as well as a stronger inhibition activity against COX-1 and COX-2 enzymes (IC50 = 52.93 ± 0.13; 89.31 ± 0.21 µg/mL).	[181]
Fahed L. et al., 2016	MRSA ATCC 33591 *S. aureus* ATCC 29213 *C. albicans* ATCC 10231 *P. aeruginosa* CIP 82118 *T. rubrum* SNB-TR1 *Tricophyton mentagrophytes* SNB-TM1 *Tricophyton violaceum* SNB-TV1 *Tricophyton tonsurans* SNB-TT	*Salvia multicaulis* Vahl	Broth microdilution assay	Zahlé EO MIC = 128 µg/mL for both S. aureus strains. Good antimicrobial activity was observed for nerolidol, the major active compound (MIC = 128 µg/mL for *S. aureus* strains and 64 µg/mL for *Tricophyton rubrum*).	[206]
Mahdavi B et al., 2017	MRSA *S. aureus* ATCC 25923 *B. thuringiensis* TACC 10792 *B. subtilis* ATCC 11774 *S. epidermidis* ATCC 1228 *E. faecalis* ATCC 14506 *A. hydrophilia* ATCC 7966 *E. coli* ATCC 10536 *E. aerogenes* ATCC 13048 *P. mirabilis* ATCC 12453 *P. vulgaris* ATCC 33420 *S. typhimurium* ATCC 51812 *S. sonnei* ATCC 29930 *S. marcescens* ATCC 13880 *V. parahaemolyticus* ATCC 17802 *P. aeruginosa* ATCC 10145 *C. albicans* ATCC 90028 *C. parapsilosis* ATCC 22019	*Etlingera sayapensis* A.D. Poulsen and Ibrahim	Disc diffusion assay MIC method	The EO obtained from the rhizome of E. sayapensis presented potent antimicrobial activity against 13 of 18 tested strains, including MRSA, *S. aureus*, *E. coli*, *P. mirabilis*, *B. subtilis*, *C. albicans*.	[207]
Gadisa E et al., 2019	MRSA *E. coli*—MDR strain *K. pneumoniae*—MDR strain *S. aureus* ATCC 25922 *E. coli* ATCC 25922 *K. pneumoniae* ATCC 700603	*Blepharis cuspidata* Qoree waraantii *Boswellia ogadensis* Vollesen *Thymus schimper* used in combination	Broth microdilution assay FIC index	The combination of *B. cuspidata* and *T. schimperi* EOs highlighted a MIC/MBC = 0.39 µg/mL against MRSA. Moreover, association of *B. cuspidata* and *B. ogadensis* had a MIC of 1.56 µg/mL for MRSA. A FICI of 0.38 (a synergistic effect) was obtained for MRSA using *B. cuspidata* + *B. orgadensis*, and of 0.25 for *K. pneumoniae* MDR, for the same association.	[208]
Bano S et al., 2020	MRSA *E. coli* *P. aeruginosa*—total drug resistant *K. pneumoniae*	*Terminalia arjuna* (Roxb.) Wight and Arn.	Not mentioned	MIC = 0.32 mg/mL for leaf oil and 0.16 mg/mL for fruit oil (against MRSA).	[209]
Ding L. et al., 2020	MRSA 134/94 R9 *S. aureus* SG 511 *P. aeruginosa* K799/61 BG137 B7 *B. subtilis* ATCC 6633 *Mycobacterium vaccae* IMET 10670 Vancomycin-resistant *E. faecalis* 1528 R10 *E. coli* SG458 *Sporobolomyces salmonicolor* SBUG 549 *C albicans* *Penicillium notatum* JP36	Geosmin compounds Compound 6: 4*β*,10*α*-eudesmane-5*β*,11-diol EO	Agar diffusion assay	The 4*β*,10*α*-eudesmane-5*β*,11-diol (compound 6) was reported as the active substance in EO from the aromatic grass *Cymbopogon distans* (an oil used in folk medicine to treat microbial infections, inflammations and colds or to protect books from insects and fungi, in ancient China). Compound 6 exhibited large antimicrobial activity against all tested strains (inhibition zone for MRSA and *P. aeruginosa* = 11 mm).	[210]
Jaradat N et al., 2017	MRSA *S. aureus* ATCC 25923 *E. coli* ATCC 25922 *P. aeruginosa* ATCC 27853 *C. albicans*	*Ruta chalepensis* L. (fringed rue)	Broth microdilution assay MIC assay	Linalyl acetate and β-linalol were determine to be the major active compounds having good antibacterial and antioxidant activities. The best antimicrobial activity was obtained for *E. coli*, *P. aeruginosa*, *S. aureus* and MRSA (MIC for R. chalepensis from Jerusalem = 4 mg/mL vs. gentamycin 1.5 mg/mL).	[211]
Man A et al., 2019	MSSA ATCC 29213, MRSA ATCC 43300 *E. faecalis* ATCC 29212 *E. coli* ATCC 25922 *K. pneumoniae* ATCC 13883 *P. aeruginosa* ATCC 27853	Micellar and aqueous extracts of EOs: *Boswellia sacra* Flueck. resin (Frankincense) *Myrtus communis* L. (common myrtle) *Thymus vulgaris* L. (common thyme) *Citrus limon* L. *Origanum vulgare* Linnaeus (oregano) *Lavandula augustifolia* Mill. (English lavender)	MIC and MBC determination methods	Good antibacterial activity was observed for oregano, thyme, lemon and lavender EOs, with Gram-positive cocci including MRSA being the most susceptible bacteria and *P. aeruginosa* being the most resistant. The best antibacterial activity was observed for oregano EO (decreased MIC/MBC ratio up to 64 times). It was observed that colloid or micelle suspensions of EOs (oregano, thyme and lemon whole EOs) may be more efficient than antimicrobial agents (in the case of MRSA).	[41]
Kot B et al., 2017	MRSA	Thyme oil *trans*-cinnamaldehyde Ferulic acid p-coumaric acid caffeic acid lavender oil geranium oil tea tree oil	Not mentioned	After 48 h of treatment, thyme oil decreased the biofilm mass by up to 85% and the metabolic activity of biofilms by up to 88.7%.	[212]
Kwiatkowski P et al., 2020	MRSA ATCC 43300	1,8-cineole eugenol carvacrol linalool linalyl acetate *trans*-anethole thymol menthone menthol β-caryophyllene	Antibiotic susceptibility testing: Kirby–Bauer method and D-test *mecA* gene detection Broth microdilution Checkerboard method FTIR spectroscopic analyses	Good anti-MRSA activity for 8 of the 10 tested EOs was observed. Thymol expressed the most powerful inhibitory activity against the tested strain. Linalyl acetate alone or especially in combination with methicillin was effective against low- and high-level beta-lactam resistant MRSA strains, effect attributed to the 2 methoxy moieties present in its molecule. Further in vivo studies are required to assess cytotoxicity and establish the safe concentration of linalyl acetate-methicillin.	[213]
Kwiatkowski P et al., 2019	MRSA (*S. aureus* ATCC 43300 mutated strains)	1,8-cineole eugenol carvacrol linalool (-)-menthone linalyl acetate *trans*-anethole versus mupirocin-susceptible (MupS) and low-level mupiocin-resistant (MupRL) MRSA	Broth microdilution Checkerboard method	Both strains of MRSA showed sensibility to the tested EOs, with carvacrol expressing the highest inhibitory activity (MIC = 0.48–0.95 mg/mL). 1,8-cineole expressed synergistic activity with penicillin G against MupRL MRSA, therefore increasing its activity against both tested strains (MupS and MupRL MRSA).	[184]
Cui H et al., 2016	MRSA ATCC 43300	Cinnamon oil encapsulated in liposomes	Broth microdilution Time-kill assay MBIC and MBEC assays MTT staining method Biofilm CFU counting Plate colony-counting method	Potent antibacterial and antibiofilm action was observed for the tested EO against MRSA, after at least 4h treatment (MIC/MBC = 0.25 mg/mL; MBIC/MBEC = 1.00 mg/mL). The bactericidal effect was highlighted by the irreversible destruction of MRSA cell membrane (rough, wrinkled and irregular). A potent antibiofilm activity was observed at 1.0 mg/mL cinnamon oil. Anti-biofilm properties of liposomes containing cinnamon oil highlighted a gradual and consecutive decrease of viable MRSA cells, depending on the conditions (stainless steel, gauze, nylon membrane, exposure time).	[214]
Perez AP et al., 2019	MRSA isolates (Cordobes clone, DOS61, DOS90 and DOS59)	*Thymus vulgaris* L. (common thyme) nanovesicular formulation	MTT assay	The antibiofilm effect was observed for nanoarchaeosomes (MIC = 4 mg/mL), this nanovesicular formulation demonstrated good stability during storage. Moreover, the macrophages (J774A.1) viability was diminished after 24 h of incubation for both formulations (including nanoliposomes) at 0.4 mg/mL.	[215]
Farias KS et al., 2019	MRSA ATCC 43300 *P. aeruginosa* ATCC 27853 *T. vaginalis* ATCC 30236 *S. epidermidis* ATCC 35984 *K. pneumoniae* clinical isolate	*Nectandra amazonum* *Nectrandra cuspidate* *Nectandra gardineri* *Nectranda hihua* *Nectandra megapotamica*	Crystal violet assay Anti-*Trichomonas vaginalis* assay	(+)-α-bisabolol (found in a concentration of 93.7% in *N. megapotamica* leaves) presented a potent antibiofilm effect against MRSA (10 µg/mL) and *P. aeruginosa* (100 µg/mL). Good anti-trichomoniasis activity was found (IC50 = 98.7 µg/mL), together with cytotoxic and haemolytic actions in Vero cells and human erythrocytes.	[216]
Eid AM et al., 2021	MRSA *P. aeruginosa* ATCC 9027 *K. pneumoniae* ATCC 13883 *E. coli* ATCC 25922 *S. aureus ATCC 25923* (*P. mirabilis?*) *C. albicans ATCC 90028*	*Coriandrum sativum* L. (coriander) nanoemulgel	Agar diffusion	Good antibacterial and anticancer effects observed for the nanoemulgel, compared with the crude coriander oil. (MRSA MIC = 6.5 µg/mL; IC50 = 28.84 µg/mL for MCF-7 (human breast cancer cells), 24.54 µg/mL for HeLa (human cervical epithelioid carcinoma cells); and 28.18 µg/mL for Hep3B (hepatocellular carcinoma cells))	[217]
Bako C et al., 2021	MRSA 4262 *P. aeruginosa* ATCC 27853	*Salvia sclarea* L. (clary sage)	Brain–heart infusion broth Thin layer chromatography without separation Post-chromatographic detection	Clary sage exhibited a 7.57 mm inhibition zone for MRSA and 7.51 mm for *P. aeruginosa*.	[218]
Bushra Jamil et al., 2016	MRSA β-lactamase producing *E. coli*	*Elettaria cardamomum* (L.) Maton (green cardamom) oil chitosan nano-particles	Kirby–Bauer disc diffusion Agar well diffusion Broth dilution	Non-cytotoxic (on human corneal epithelial cells) and non-haemolytic effects were observed for the cardamom oil loaded in chitosan nano-particles, as well as potent antibacterial properties against the tested strains.	[219]
Khoury M et al., 2019	MRSA ATCC 33591 *E. coli* ATCC 25922 *S. aureus* ATCC 29213 *C. albicans* ATCC 10231 *C. parapsilosis* ATCC 22019 *Cryptococcus neoformans* ATCC SNB-CN1 *Trichophyton rubrum* SNB-TR1 *T. violaceum* *T. soudanense* SNB-TS1 *T. tonsurans* SNB-TT1 *T. mentagrophytes* SNB-AF1 *Aspergillus fumigatus* ATCC SNB-AF1	*Hirtellina lobelia* DC.	Broth microdilution method Checkerboard assay	Good bactericidal effects against *S. aureus* strains, including MRSA (MIC/MBC = 128 µg/mL). Synergism was observed in association with vancomycin (against *S. aureus*). The main compound, α-bisabolol, was found to have potent antimicrobial potential (including anti-fungal).	[220]
Viktorova J et al., 2020	MRSA DBM 12 S. aureus ATCC 25923 *Salmonella enterica* CCM 4420 *Proteus vulgaris* DBM 3022 *Mycobacterium smegmatis* ATCC 70084 *P. aeruginosa* CCM 3955 *C. albicans* DBM 2186 *C. famata* DBM 23 Cryptococcus albidus DBM 4	*Cymbopogon citratus* (DC.) Stapf (lemon grass)	Broth microdilution Autoinducer bioassay Pgp-Glo assay Static antibiofilm assay Resazurin assay Mature biofilm assay	Citral (the main component of lemongrass EO—63%) was almost 100 times more active than lemongrass EO. Both disrupted bacterial communication and adhesion during biofilm formation (in *S. aureus* and *P. aeruginosa*), with citral having the highest effect (citral IC50 was 7–70 times lower that lemongrass IC50). Citral had 5 times more potent antibiofilm activity on *P. aeruginosa* than on MRSA. Lemongrass EO (and not citral alone) induced a sensitizing action on MRSA and on ovarian carcinoma cells resistant to doxorubicin, probably through the inhibition of P-glycoprotein efflux pump.	[221]
Manzuoerh R et al., 2019	MRSA clinical isolate Methicillin-sensitive *S. aureus* ATCC 25923 Methicillin-resistant *S. aureus* ATCC 33591	*Anethum graveolens* L. (dill)	Broth nutrition Wound model	Dill EO application on wounds inhibited bacterial growth and diminished wound area compared to the control group. It reduced the inflammatory process (by decreasing p53 and caspase-3 expression) and enhanced re-epithelization, angiogenesis, collagen and fibroblast deposition after topical administration. The expression of Bcl-2, p53 caspase-3, VEGF and FGF-2 was increased in the group treated with the EO.	[185]
Taha AM et al., 2017	MRSA RCMB 2658 *E. coli* RCMB 010052 *Geotrichum candidum* RCMB 05097 *P. aeruginosa* RCMB 010043 *B. subtilis* RCMB 010067 *H. pylori* RCMB 088452 *A. fumigatus* RCMB 02568 *M. tuberculosis*	*Cinnamomum glanduliferum* (Wall) Nees (false camphor tree)	Agar-well diffusion Resazurin microtiter assay Broth microdilution	The antibacterial activity of bark EO was good against MRSA (MIC = 7.81 µg/mL) and strong against *E. coli* (activity index = 1 and MIC = 0.49 µg/mL). Toxicity against colon (HCT-116), liver (HepG2) and breast (MCF-7) carcinoma cell lines was observed (IC50 = 9.1; 42.4; and 57.3 µg/mL). It was suggested that the antimicrobial and cytotoxic effects were due to eucalyptol (65.87%), as well as terpinene-4-ol (7.57%), α-terpineol (7.39%) and others.	[222]
Jaradat N et al., 2016	MRSA *S. aureus* ATCC 25923 *E. coli* ATCC 25922 *P. aeruginosa* ATCC 27853 *C. albicans*—clinical isolate *Pheretima posthuma*	*Thymus bovei* Benth.	MIC assay Broth microdilution Antihelmintic assay	Trans-geraniol (35.38%), α-citral (20.37%), β-citral (14.76%) and cis-geraniol (7.38%) were the major identified phytocompounds (MRSA and *E. coli* MIC = 0.5 mg/mL). The most potent antimicrobial activity was observed in the case of *P. aeruginosa*, *S. aureus* and *C. albicans* (MIC = 0.25 mg/mL). Strong antihelmintic activity was observed for the tested EO (compared with the piperazine citrate reference standard).	[223]
Lahmar A et al., 2016	MRSA 138; 760; 753 ESBL *E. coli* Ceftazidime-resistant *A. baumannii*	*Pituranthos chloranthus* *Teucruim ramosissimum* *Pistacia lentiscus* alone and in combination with ABs	Broth microdilution Broth microdilution checkerboard Time-kill assay	MRSA MIC values varied as follows: 0.25–0.5 mg/mL for *P. chloranthus* EO; 0.25–1 mg/mL for *T. ramosissimum*; and 0.125–1 mg/mL for *P. lentiscus*. MIC values for *E. coli* and *A. baumannii* were found to be higher. Higher antibacterial effect against *E. coli* (synergic action) was observed when EOs were associated with ofloxacin and novobiocin (MIC was reduces up to 64 times). EOs enhanced the effect of all ABs used for MRSA strains (especially for MRSA 760).	[224]
Shehadeh M et al., 2019	*S. aureus* ATCC 25923 MRSA *E. faecium* ATCC 700221 *E. coli* ATCC 25922 *P. aeruginosa* ATCC 27853	*Origanum syriacum* L. (bible hyssop)	Broth microdilution	The most effective EOs against MRSA and other *S. aureus* strains were those rich in thymol (MIC = 390 µg/mL). The chemotypes rich in α-terpinene were effective against P. aeruginosa (MIC = 1560 µg/mL) and EOs rich in gamma-terpinene expressed the highest antibacterial properties against *E. faecium* (MIC = 97 µg/mL), as well as good antioxidant effect.	[225]
Demirci F et al., 2018	MRSA ATCC 700699 *S. aureus* ATCC BAA-1026 *S. epidermidis* ATCC 14990 *S. pyogenes* ATCC 19615 *S. pneumoniae* ATCC 10015 *P. aeruginosa* ATCC 10145 *H. influenzae* ATCC 49247 *M. catarrhalis* ATCC 23245	*Thymus sipyleus* Boiss.	Agar diffusion Broth microdilution Vapour diffusion	The best antibacterial effect for thyme scented lemon was noticed against *S. aureus*, *S. pyogenes* and *M. catarrhalis* (MIC = 12–13 µg/mL). MRSA MIC = 310 µg/mL and *P. aeruginosa* MIC = 1250 µg/mL (lowest inhibitory effect). The anti-inflammatory effect was 12.1 ± 1.8% in 100 µg/mL, both effects being required for efficient inhalations in the treatment of rhinosinusitis.	[226]
Mahboubi M et al., 2016	MRSA	*Oliveria decumbens* vent *Pelargonium graveolens* L’Hér. vs. mupirocin	Skin wound infection in mice	Similar potent antibacterial effects against MRSA were obtained for both mupirocin and the herbal cream containing den EO (from *Oliveria decumbens* aerial part) and geranium EO (from *Pelargonium graveolens* leaves), with log CFU = 2.46 ± 0.32 and 2.5 ± 0.26, respectively, compared to 5.9 ± 0.26 and 5.65 ± 0.23 for placebo and control groups.	[186]
Salameh N et al., 2020	MRSA *P. mirabilis* *S. aureus* clinical isolates *E. coli* ATCC 25922 *E. faecium* ATCC 700221 *K. pneumoniae* ATCC 13883 *P. aeruginosa* ATCC 27853 *Shigella sonnei* ATCC 25931 *S. aureus* ATCC 25923 *C. albicans* ATCC 90028 *Epidermophyton floccosum* ATCC 52066	*Micromeria fruticosa serpyllifolia*	Broth microdilution Agar dilution	Low antimicrobial activity against MRSA (MIC = 3.125–6.250 mg/mL). The EO samples from different parts of Palestine exhibited different antimicrobial and antioxidant effects.	[227]
Jaradat N et al., 2019	MRSA *S. aureus* ATCC 25923 *E. faecium* ATCC 700221 *E. coli* ATCC 25922 *P. aeruginosa* ATCC 27853 *Shigella sonnie* ATCC 25931 *C. albicans* ATCC 90028 *Epidermophyton floccosum* ATCC 10231	*Stachys viticina* Boiss.	Microdilution assay	The strongest antibacterial activity of EO from leaves was observed against MRSA (MIC = 0.039 mg/mL). Cytotoxicity was observed for the 2 cancer cell lines: HeLa (cervical adenocarcinoma, with a 95% inhibition at 1.25 mg/mL) and Colo-205 (colon, with a 90% inhibition at 0.5 mg/mL). High COX1,2 inhibitory activity (similar to that of NSAID etodolac), as well as antioxidant activity (IC50 = 19.95 µg/mL), were determined.	[228]
Kwiatkowski P et al., 2019	MRSA S. aureus ATCC 43300 (control)	*Lavandula augustifolia* Mill. associated with octenidine dihydrochloride	Broth microdilution Checkerboard assay Time-kill curve assay	Lavender EO (flowering herb) showed activity against MRSA (MIC = 13.72 µg/mL). Synergistic activity observed with octenidine dihydrochloride (FICI = 0.11–0.26). Lavender EO appears to modify the bacterial cell wall structure and might be used for enhancing the activity of conventional antiseptics.	[229]
Chen J et al., 2020	MRSA ATCC 43300 *S. aureus* ATCC 25923 *E. faecalis* ATCC 29212 *B. subtilis* ATCC 21332 *P. aeruginosa* ATCC 27853 *S. gallinarum* CVCC 79207 *E. coli* ATCC 25922	*Cinnamomum camphora* (Linn.) Presl (camphor)	Broth microdilution Field emission scanning electron microscopy Transmission electron microscopy	EO showed a certain activity against MRSA (MIC = 0.8 mg/mL, MBC = 1.6 mg/mL) and good antibacterial effects on the other strains, dependent on the concentration.	[187]
Noumi E et al., 2018	MRSA—28 strains *S. aureus* ATCC 6538 *S. aureus* ATCC 4330 *P. aeruginosa* *C. violaceum* ATCC 12 472	*Melaleuca alternifolia* (Maiden and Betche) Cheel (tea tree) Terpinen-4-ol	Disc diffusion assay Microdilution assay Semi-quantitative adherence assay Crystal violet assay Violacein inhibition assay Swarming inhibition assay	Sa 442 gene was identified in all confirmed 28 MRSA strains (mecA gene positive, MIC = 0.048–3.125 mg/mL for tea tree EO and 0.048–1.52 mg/mL for the terpinen-4-ol; MBC = 25–50 mg/mL for tea tree EO and 6.25–50 mg/mL for terpinen-4-ol). Both tea tree EO and terpinene-4-ol exhibited the adhesivity of MRSA on polystyrene (MIC/16 = 0.003 mg/mL). Terpinen-4-ol showed anti-biofilm activity of 73.70%, while low concentrations of tea tree EO inhibited the formation of biofilm and cell communication.	[230]
Gradinaru AC et al., 2018	*P. aeruginosa* ATCC 27853 *S. aureus* ATCC 25923 *S. pneumoniae* ATCC 49619 Penicillin-resistant *S. pneumoniae* (ARPA 2351) S. aureus (MRSA 37, 4185) S. pneumoniae (PRSP 4423, 4546, 4566)	*Trachyspermum ammi* (L.) Sprague ex Turrill (ajwain, ajowan)	Broth microdilution Checkerboard method	A synergistic action of ajowan EO/thymol + amoxicillin was observed on MRSA (FICI = 0.37–0.50). The same effect was observed for the association of EO + ciprofloxacin in the case of *P. aeruginosa*, *S. aureus* and penicillin-resistant *S. pneumoniae* (FICI = 0.37–0.5).	[231]
Marino A et al., 2020	*S. aureus* ATCC 6538 *S. aureus* ATCC 43300 *S. epidermidis* ATCC 35984 *L. monocytogenes* ATCC 13932 *B. subtilis* ATCC 6633 *S. aureus* 7786 MRSA (*S. aureus* 815) *S. aureus* 74CCH-MRSA *P. aeruginosa* ATCC 9027 Candida sp.	*Coridothymus capitatus* (L.) Reichenb. fil. Hydrolate alone or in association with tetracycline/itraconazole	Checkerboard method Broth microdilution Propidium iodide and MitoTracker staining	Spanish oregano (also known as *Thymus capitatus* (L.) Hoffmanns. and Link) EO obtained from flowers was used. Antimicrobial activity of the prepared hydrolates (alone or in combination with tetracyline and itraconazole) was assessed. The hydrolate exhibited good antimicrobial activity, as well as a synergistic action (alteration of mitochondrial function) with itraconazole against *C. krusei* and an additive effect (alteration of membrane permeability) with tetracycline against MRSA strains.	[232]
Tadic V et al., 2017	MRSA clinical strain MSSA ATCC 29213 *E coli* ATCC 25922 *K pneumoniae* cabapenem susceptible *K pneumoniae* cabapenem resistant *C albicans* ATCC 14053	*Sideritis romana* L. subsp. *purpurea* (Tal. ex Benth.) Heywood (purple ironwort)	Mueller Hinton broth	Forty-three potentially active compounds were identified, the most abundant being bicyclogermacrene (23.8%), germacrene F (8%), (*E*)-caryophyllene (7.9%) and spathulenol (5.5%). High activity was noticed against both MSSA (MIC = 0.307 mg/mL, MBC = 0.615 mg/mL) and MRSA (MIC = 0.307 mg/mL and MBC = 0.153 mg/mL), at high and low inoculum. The same was observed for both extracts in 1.2-dichloroethane and methanol.	[233]
Ramirez-Rueda RY et al., 2019	MRSA ATCC 43300 *E faecalis* vancomycin-resistant ATCC 51299	*Chrysopogon zizaniodes* (L.) Roberty known as *Vetiveria zizanioides* (L.) Nash (vetiver grass)	Mueller Hinton broth	The root extract EO showed activity against MRSA (MIC = 62.5 µg/mL) and VREF (MIC = 125 µg/mL). Cedr-8-en-13-ol was proposed as the most important compound exhibiting antimicrobial activity.	[234]
Brun P et al., 2019	MRSA *P aeruginosa* *C glabrata* *H simplex* virus type 1 strain 16	*Melaleuca alternifolia* (Maiden and Betche) Cheel (tea tree)	Broth microdilution	Ten commercially available tea tree EO products were tested against strains grown in planktonic mode or biofilms. Regarding MRSA, MIC varied from 0.027–2.5% *v*/*v* for the tested substances. The authors concluded that the antimicrobial activity could not be attributed to terpinene-4-ol alone and stipulated that it is a consequence of synergism among different constituents of the EOs.	[235]
Jaradat NA et al., 2016	*S. aureus* ATCC 25923 *E coli* ATCC 25922 *P aeruginosa* ATCC 27853 MRSA *C. albicans* clinical isolates	*Trichodesma africanum* (L.) Sm.	Broth microdilution	MRSA MIC = 3 mg/mL. The microwave-ultrasonic extraction technique yielded the best results.	[236]
Saidi M et al., 2016	MRSA E coli ESBL producing P aeruginosa MBL producing	*Thymus daenensis* Celak.	Disc diffusion	MRSA MIC: 25 mg/mL. Good cyto-tolerability, as well as antioxidant properties were found.	[237]
Wang B et al., 2017	MRSA ATCC 43300 MRSA ATCC 33591 *E. coli* ATCC 25922 *E. coli* ATCC 44102 *S. aureus* ATCC 25923 *S. aureus* ATCC 26003	Pogostone—from *Pogostemon cablin* (Blanco) Beneth. (patchouli)	Broth microdilution	Molecular docking studies of pogostone (obtained from patchouli EO) with pentaerythritol tetranitrate reductase were performed and the structure–activity relationship was analysed. Compound 3h exhibited the highest antimicrobial activity against MRSA (MIC = 8 µg/mL), similar to that of levofloxacin and vancomycin that were used as positive controls.	[238]
*S. aureus*—vancomycin resistant					
Vasconcelos SECB et al., 2017	VRSA (*S aureus* strains isolated from food) and oxacillin resistant S aureus S aureus ATCC 6538	*Plectranthus amboinicus* Lour. (Mexican mint)	Disc diffusion Microdilution Microtiter-plate technique Crystal violet assay Counting viable cells	EO obtained from the leaves and stem was used to evaluate the antimicrobial and antibiofilm activity. Carvacrol was determined as the major component in the EO. All tested strains were sensitive to carvacrol and EO, and the best activity (no viable cells on the biofilm) was noticed for the combination of both products (MIC = 0.5 mg/mL).	[189]
